# Rapid and profound decay of inducible and intact HIV genomes in early-treated Thai children

**DOI:** 10.1172/JCI198054

**Published:** 2026-04-02

**Authors:** Marta Massanella, Caroline Dufour, Amélie Pagliuzza, Audrée Lemieux, Corentin Richard, Jintanat Ananworanich, Louise Leyre, Thidarat Jupimai, Supranee Buranapraditkun, Rapisa Nantanee, Julie L. Mitchell, Panadda Sawangsinth, Mark de Souza, Piyarat Suntarattiwong, Suparat Kanjanavanit, Pope Kosalaraksa, Thitiporn Borkird, Witaya Petdachai, Kulkanya Chokephaibulkit, Lydie Trautmann, Rémi Fromentin, Thanyawee Puthanakit, Nicolas Chomont, on behalf of the HIV Netherlands Australia Thailand Research Collaboration 209 and 194 (HIVNAT209 and HIVNAT194) study groups

**Affiliations:** 1 Centre de Recherche du CHUM and Department of Microbiology, Infectiology and Immunology, Université de Montréal, Montréal, Quebec, Canada.; 2 Department of Global Health, Amsterdam Medical Center, University of Amsterdam, Amsterdam, Netherlands.; 3 Center of Excellence in Pediatric Infectious Diseases and Vaccines, Chulalongkorn University, Bangkok, Thailand.; 4 Division of Allergy and Clinical Immunology, Department of Medicine, King Chulalongkorn Memorial Hospital, Faculty of Medicine, Chulalongkorn University, Thai Red Cross Society, Bangkok, Thailand.; 5 Center of Excellence in Thai Pediatric Gastroenterology, Hepatology and Immunology (TPGHAI);; 6 Center of Excellence in Vaccine Research and Development (Chula Vaccine Research Center-Chula VRC); and; 7 Department of Pediatrics, Faculty of Medicine, Chulalongkorn University, Bangkok, Thailand.; 8 Vaccine and Gene Therapy Institute, Oregon Health & Science University, Beaverton, Oregon, USA.; 9 SEARCH, Thai Red Cross AIDS Research Centre (TRC-ARC), Bangkok, Thailand.; 10 Queen Sirikit National Institute of Child Health, Bangkok, Thailand.; 11 Nakornping Hospital, Chiang Mai, Thailand.; 12 Srinagarind Hospital, Khon Kaen University, Khon Kaen, Thailand.; 13 Hat Yai Hospital, Songkhla, Thailand.; 14 Phrachomklao Hospital, Phetchaburi, Thailand.; 15 Department of Pediatrics, Faculty of Medicine, Siriraj Hospital, Mahidol University, Bangkok, Thailand.; 16 U.S. Military HIV Research Program, CIDR, Walter Reed Army Institute of Research, Silver Spring, Maryland, USA.; 17 Henry M. Jackson Foundation for the Advancement of Military Medicine, Bethesda, Maryland, USA.; 18 HIV-NAT, TRC-ARC, Bangkok, Thailand.; 19 The HIVNAT209 and HIVNAT194 study groups are detailed in Supplemental Acknowledgments.

**Keywords:** AIDS/HIV, Immunology, Virology, T cells

## Abstract

Early initiation of antiretroviral therapy (ART) in perinatally HIV-infected children significantly limits the establishment of the viral reservoir. However, the long-term impact of this intervention remains unclear. We measured the frequency of inducible, translation-competent, and replication-competent proviruses in samples from 62 children who initiated ART early and remained virally suppressed for up to 9.9 years. Only a small fraction of HIV genomes produced HIV transcripts, viral proteins, or infectious virions. Accordingly, replication-competent virus was detected in only 11% of the participants. Despite the predominance of naive cells in pediatric blood, most proviruses were detected in memory CD4^+^ T cells, especially central memory cells. Longitudinal analysis revealed a biphasic decay in HIV DNA: an initial decline followed by long-term stability, which was associated with extensive expansions of infected T cell clones. In contrast, inducible proviruses declined continuously and became undetectable in most children after 5 years. Near full-length sequencing of 1,305 HIV genomes revealed a dramatic reduction in genetically intact proviruses, from pre-ART to after 7 years of ART. Together, these findings suggest that the intact viral reservoir rapidly decays in early-treated children, offering critical insights for pediatric HIV cure strategies.

## Abstract 2

Early ART in perinatally HIV-infected children leads to near-complete clearance of intact proviruses, supporting cure potential in pediatrics.

## Graphical abstract

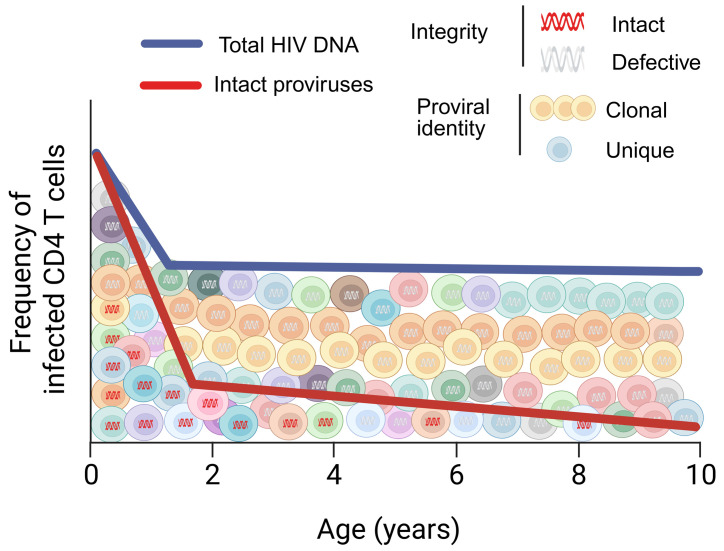

## Introduction

HIV persists in viral reservoirs despite prolonged antiretroviral therapy (ART) in both adults with acquired infection and children born with HIV (1–4). Early initiation of ART is the only intervention to date that has demonstrated a significant impact on reducing the size of these reservoirs. Multiple studies have demonstrated that starting ART during the first days or weeks of infection reduces the frequency of persistently infected cells in children after perinatal infection (5–14). Furthermore, both our work and that of others have shown that early ART initiation provides long-term benefits, maintaining very low frequencies of HIV-infected cells even after years of suppressive therapy (15–18). Most of these studies have relied on HIV DNA quantification as a proxy for the size of the HIV reservoir. However, this approach often overestimates the true size of the reservoir, as many of the proviruses that persist in people with HIV on ART harbor large deletions or other genetic defects that preclude the production of replication-competent viruses — the primary source of viral rebound upon ART interruption (19, 20). Only a limited number of studies have examined the genetic integrity of the viral reservoir in pediatric populations (6, 21–24). Collectively, these works suggest that, similar to adults, most proviruses in children are defective. Additionally, intact proviruses tend to decay more rapidly in children, possibly due to immune pressures, including NK cells and other innate immune responses (25–27). This was exemplified in a recent study of twins who acquired HIV perinatally, started ART at week 10 after birth, and remained on ART for 28 years, in whom intact proviruses were exceedingly rare (28). This rapid decay of the HIV reservoir in children who acquired HIV perinatally may reflect a combination of factors, including early ART initiation, enhanced innate immune responses, and potentially unique mechanisms of HIV persistence in children that differ from those observed in adults.

Whether the mechanisms of HIV persistence are similar in adults and children remains unclear. Like adults, it is unlikely that the viral reservoir in children is replenished by ongoing low-level replication during ART (29). Instead, clonal expansion of cells harboring intact HIV genomes likely contributes to the maintenance of the viral reservoir in children on ART (6, 21, 30). As in adults, HIV-infected cell clones in children are established early, can persist for years on ART, and may be driven by proviral integration into proto-oncogenes, such as BACH2 and STAT5B (31).

Intact HIV genomes in children on ART not only are rare but also may display lower inducibility, as evidenced by low frequencies of replication-competent HIV detected in viral outgrowth assay (VOA) or other inducibility assays, such as the Tat-Rev induced limiting dilution assay (TILDA), compared with adults (15, 22, 32). The virological and immunological mechanisms responsible for the reduced inducibility of intact HIV genomes in virally suppressed children remain poorly understood. It is possible that CD4^+^ T cells in children are less responsive to ex vivo stimuli or that HIV proviruses persist in cells that are more refractory to T cell activation. While memory CD4^+^ T cells are the primary reservoir for HIV in adults (33), the cellular reservoirs in children are less well characterized. Although studies in infant rhesus macaques have identified naive T cells as an important reservoir for SIV in ART-suppressed infants (34), human data suggest that memory cells may also be a key reservoir for HIV in children, though these analyses have been limited to a small number of participants (8, 35). In addition, whether naive or memory cells are the main contributors to HIV persistence after prolonged ART in children who initiated therapy early remains unknown.

Given the scarcity of intact HIV genomes in children on ART, and the challenges in studying these rare cells, understanding their inducibility and replication competency has been difficult. To our knowledge, no study has yet simultaneously investigated both the frequency of genetically intact HIV genomes and their inducibility in the same children on ART. In this study, we combined several virological assays to assess the inducibility, cellular distribution, and genetic integrity of HIV genomes in longitudinal samples from Thai children with perinatal HIV followed for up to 9.9 years (*N* = 62, Table 1 and Supplemental Table 1; supplemental material available online with this article; https://doi.org/10.1172/JCI198054DS1).

## Results

### Low frequencies of inducible proviruses in early-treated children on ART.

**Figure 1:**
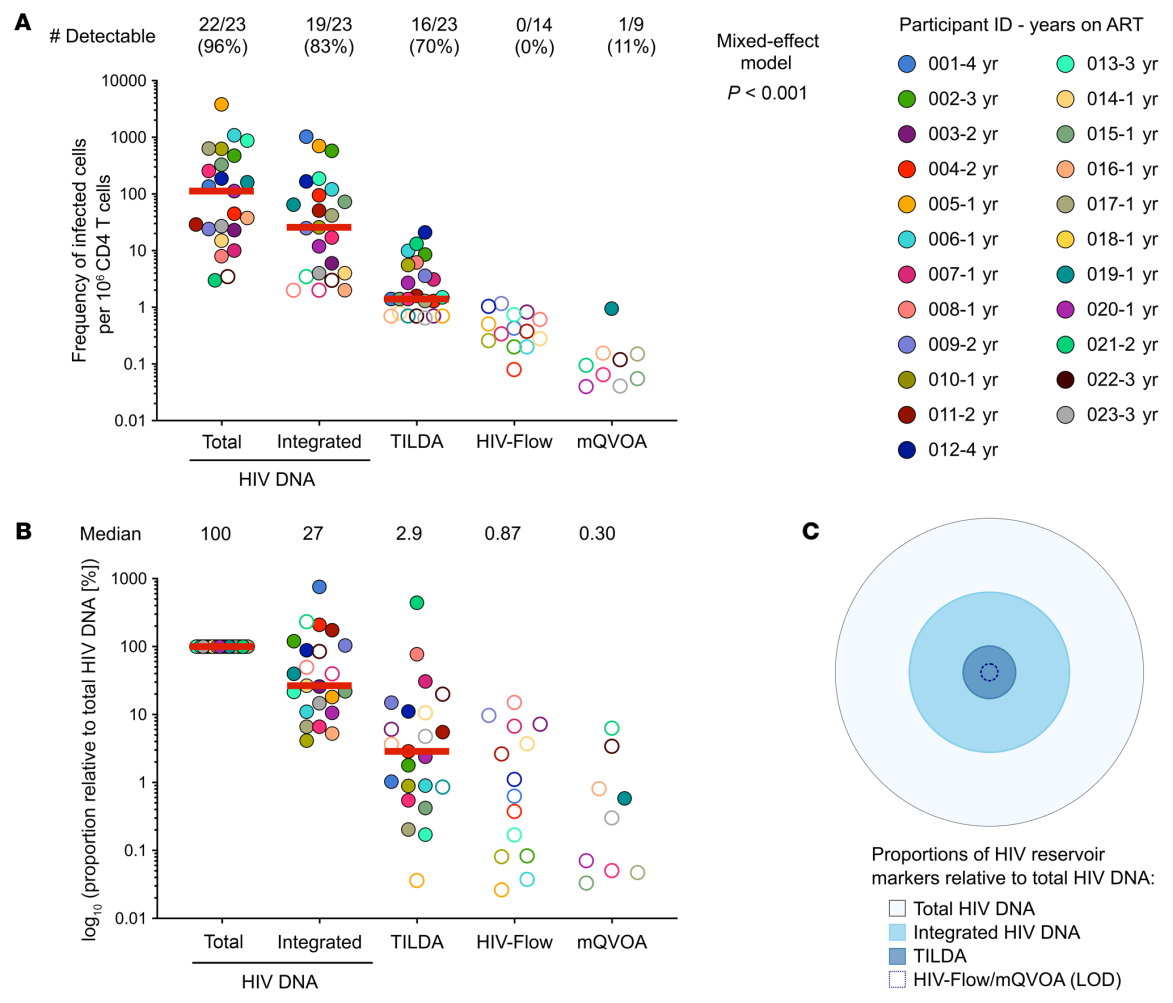
Markers of HIV persistence in early-ART-treated Thai children. (**A**) Frequencies of CD4^+^ infected cells in samples from 23 ART-suppressed children were estimated using up to 5 assays: total HIV DNA (*N* = 23), integrated HIV DNA (*N* = 23), TILDA (*N* = 23), HIV-Flow (*N* = 14), and mQVOA (*N* = 9). Undetectable samples are plotted as half of limit of detection (LOD) and represented as unfilled symbols. The total number of detectable samples and corresponding frequencies are indicated. Data were log_10_-transformed, and mixed effects analyses were performed to account for repeated measures within individuals and to determine differences between measurements. (**B**) Relative proportions of each HIV reservoir marker, normalized to the total HIV DNA levels for each individual. For panels **A** and **B**, red lines represent medians. Each participant is represented by a unique color and unique ID, followed by the number of years on suppressive ART. Undetectable measurements are represented as unfilled symbols. (**C**) Proportions of HIV reservoir markers relative to total HIV DNA are presented as a concentric circle plot. The plot uses the median values from **B** to represent the proportions.

While it is well established that ART initiation during the first weeks of life restricts the pool of cells harboring HIV DNA, little is known about the capacity of these proviruses to produce transcripts, proteins, and infectious virions. We used a combination of assays to measure the frequencies of infected cells carrying inducible viral genomes in samples from 23 ART-suppressed children who initiated ART at a median age of 9.9 [IQR 4.7–12.6] weeks and remained on ART for a median of 1.4 [1.0–2.6] years (Supplemental Table 2). We measured the levels of total and integrated HIV DNA (36), as well as the frequencies of cells harboring transcription-competent (TILDA, *N* = 23), translation-competent (HIV-Flow, *N* = 14), and replication-competent proviruses (modified Quantitative Viral Outgrowth Assay, mQVOA; *N* = 9, Supplemental Figure 1A). The median frequencies of cells harboring total and integrated HIV DNA were 113 [23–476] and 26 [4–121] per 10^6^ CD4^+^ T cells, respectively (Figure 1A). In contrast with total and integrated HIV DNA, which were readily detected in most participants (96% and 83%, respectively), CD4^+^ T cells producing multiply spliced viral transcripts following stimulation were detected in only 70% of the samples and at low frequencies (1.4 [0.7–5.6] per 10^6^ CD4^+^ T cells). Strikingly, CD4^+^ T cells capable of producing HIV proteins were not detected in these participants, despite the relatively large number of cells assayed in this pediatric population (a total of 22.5 × 10^6^ CD4^+^ T cells obtained from 14 participants, median cells tested: 1.23 × 10^6^ [0.71–1.91]). Importantly, p24^+^ cells were readily detected in CD4^+^ T cells from children who experienced virological failure as well as in virally suppressed adults (Supplemental Figure 1, B–E), demonstrating the unique nature of the pediatric reservoir. Accordingly, despite a relatively large number of cells tested (total cells tested 46.4 × 10^6^ cells, median 5.3 × 10^6^ [2.9–6.3] CD4^+^ T cells tested per individual), replication-competent HIV was detected in only 1 of the 9 samples tested (11%), with exceedingly low measures of infectious units measured. As expected, there were significant differences between the reservoir measures (mixed-effect model, *P* < 0.001), with measures of inducible reservoirs often below the limit of detection. Longitudinal measures performed on samples from 3 participants confirmed these observations (Supplemental Figure 2). The frequencies of total and integrated HIV DNA strongly correlated with each other (Spearman’s *r* = 0.74, *P* < 0.001, Supplemental Figure 3A) but not with the frequencies of cells carrying inducible viral genomes as measured by TILDA (Supplemental Figure 3, B and C).

The relative proportions of each HIV reservoir marker normalized to the total HIV DNA levels revealed that only 2.9% of all HIV genomes produced multiply spliced transcripts upon activation (Figure 1, B and C), whereas less than 1% produced viral proteins and less than 0.3% produced infectious virions. Together, these results highlight the limited inducibility and replication capacity of the viral genomes in these ART-suppressed children.

### Distribution of integrated HIV DNA in CD4^+^ T cell subsets.

**Figure 2:**
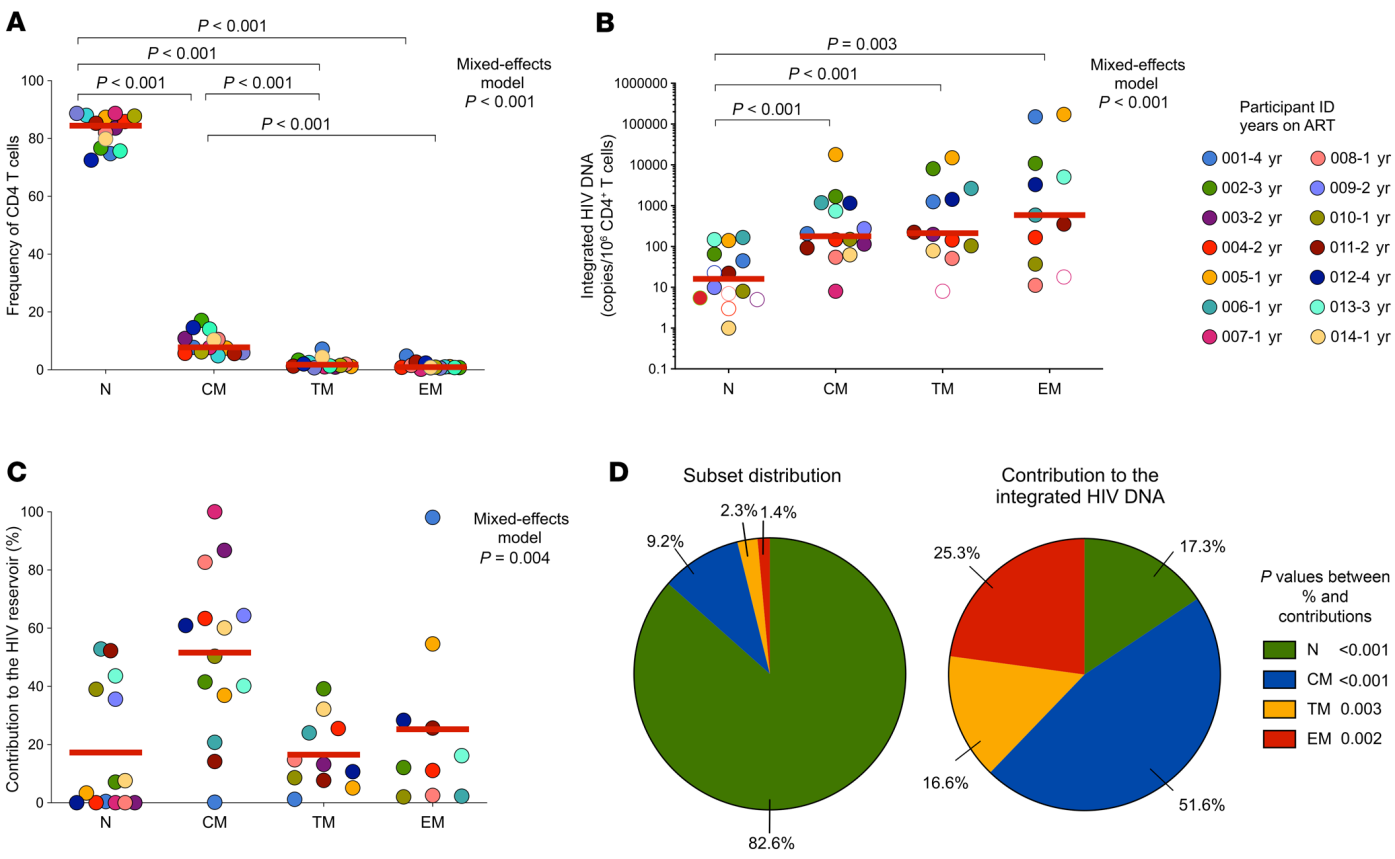
Distribution of integrated HIV DNA in CD4^+^ T cell subsets in early-ART-treated Thai children. (**A**) Percentage of CD4^+^ T cell subsets (naive, central memory [CM], transitional memory [TM], and effector memory [EM]) among total CD4^+^ T cells in 14 early ART-treated children. (**B**) Frequency of cells harboring integrated HIV DNA in sorted CD4^+^ T cell subsets from the same individuals. Undetectable measurements are represented as unfilled symbols, and the half of the LOD are plotted. (**C**) Contribution of each CD4^+^ T cell subset to the pool of HIV-infected cells from each individual. In panels **A** and **B**, red lines denote median values, whereas in panel **C**, they denote mean values. Each sample is represented by a unique color-coded symbol and unique ID, followed by the number of years on suppressive ART. Data were log_10_-transformed, and mixed effects analyses were performed, followed by Tukey’s test to identify differences between the CD4^+^ T cell subsets. PMA/ionomycin-stimulated cells and unstimulated cells are plotted as circles and squares, respectively. (**D**) Comparison of the contribution of the percentage of CD4^+^ T cell subsets to the pool of circulating CD4^+^ T cells and to the pool of HIV-infected cells, summarized by pie charts displaying the mean proportions across individuals. Wilcoxon’s matched pairs signed-rank test was used to compare the contribution of each CD4^+^ T cell subset to the pool of circulating cells with its contribution to the pool of HIV-infected cells.

To determine if the low inducibility of the viral genomes retrieved from these children could be attributed to their cellular location, we aimed to identify the cellular subsets carrying these poorly inducible proviruses. CD4^+^ T cells from 14 early-treated children on ART for a median of 2.1 [1.4–3.4] years (Supplemental Table 3) were analyzed by sorting naive, central, transitional, and effector memory cells (N, CM, TM, and EM, respectively) to quantify integrated HIV DNA within each subset (Supplemental Figure 4A). Since this experiment was combined with the HIV-Flow measures presented in Figure 1A, CD4^+^ T cells were prestimulated before sorting. Of note, stimulated and unstimulated samples showed similar distributions of CD4^+^ T cell subsets and comparable frequencies of integrated HIV DNA across subsets, indicating that short-term stimulation did not substantially affect these measurements (Supplemental Figure 4, B and C). As expected, the CD4^+^ compartment was primarily composed of naive cells (median 84.5% [76.5–87.9] naive CD4^+^ T cells), while CM, TM, and EM represented only 7.8% [5.9–11.7], 1.8% [1.2–2.7], and 0.9% [0.7–1.7] of all CD4^+^ T cells, respectively (mixed effects models *P* < 0.001, Figure 2A). All 3 memory subsets displayed higher frequencies of cells with integrated HIV DNA compared with naive cells (mixed effects models, *P* < 0.001, Figure 2B), in line with previous reports in adults (33). The median frequencies of cells harboring integrated HIV DNA increased with T cell differentiation: naive 16 [5–84], CM 179 [85–1,158], TM 226 [105–2,664], and EM 1,953 [135–45,962] infected cells per 10^6^ cells. We calculated the relative contribution of each subset to the pool of infected cells and observed that CM cells were the main contributors to the pool of cells harboring integrated HIV DNA (52% [SD 28], Figure 2C). While naive cells represented a mean of 82.6% (SD 5.72) of all CD4^+^ T cells, their contribution to the pool of cells harboring integrated HIV DNA was only 17.3% (SD 21.8) (*P* < 0.001, Figure 2D). Conversely, effector memory cells were rare (mean 1.4%) (SD 1.2) but encompassed 25.3% (SD 30.2) of cells with integrated viral genomes (*P* < 0.001, Figure 2D). These results indicate that while naive cells are abundant, they minimally contribute to the HIV reservoir and that the CM subset serves as a major reservoir for HIV in these early-treated children.

The distribution of integrated HIV DNA across T cell subsets in viremic children (Supplemental Table 4) mirrored that observed in those with viral suppression, with memory subsets — particularly CM cells — harboring the most HIV genomes (Supplemental Figure 4, D–I).

**Figure 3:**
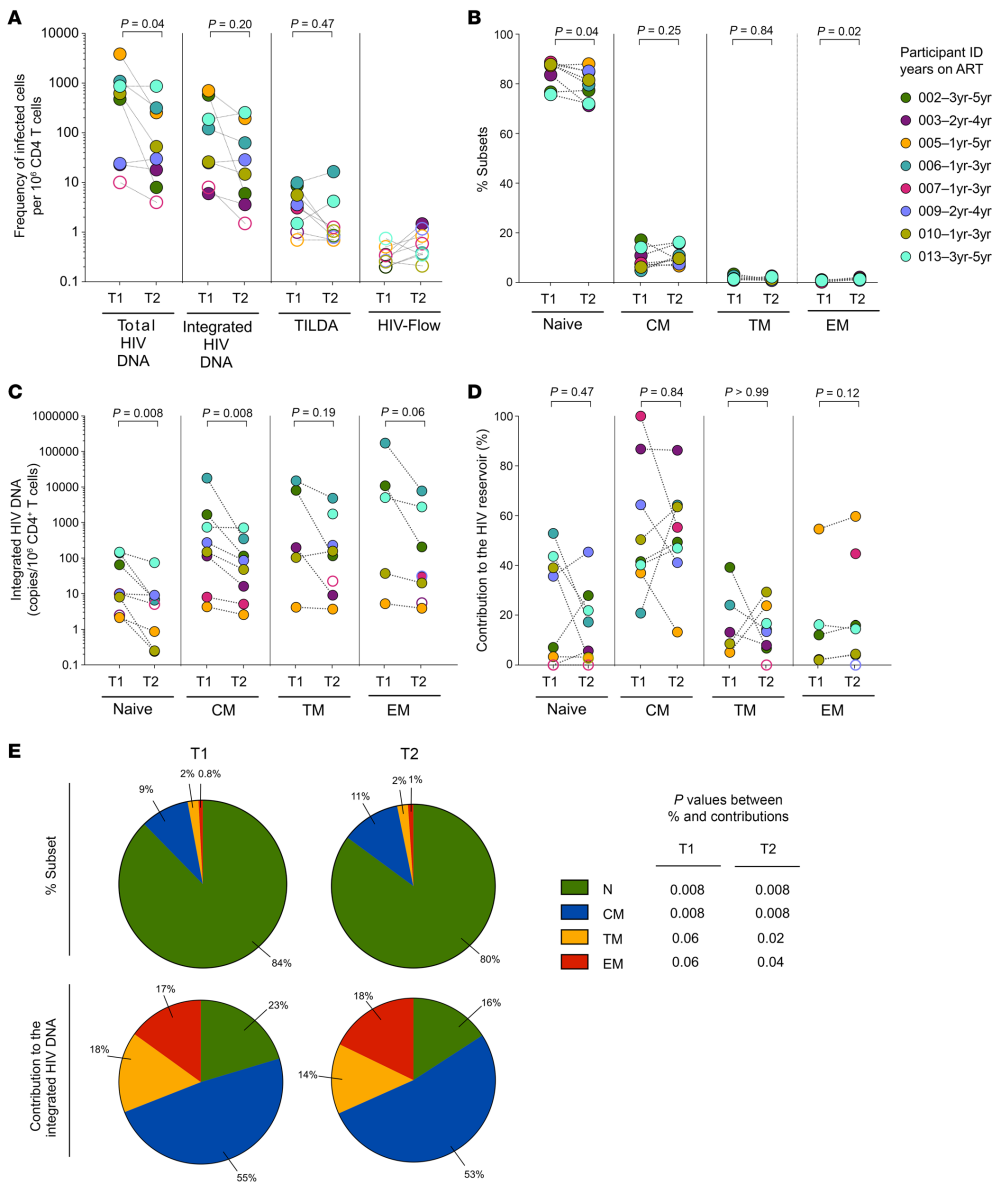
Evolution of the HIV persistence markers and distribution of integrated HIV DNA in CD4^+^ T cell subsets over time in early-ART-treated Thai children. (**A**) Changes over time in the frequencies of infected cells in samples from 8 ART-suppressed children were assessed at 2 time points using 4 assays: total HIV DNA, integrated HIV DNA, TILDA, and HIV-Flow. Undetectable measurements are represented as unfilled symbols, and the half of the LOD are plotted. (**B**) Percentage of CD4^+^ T cell subsets (naive, central memory [CM], transitional memory [TM], and effector memory [EM]) among total CD4^+^ T cells in the same 8 early-ART-treated children at the same time points. (**C**) Frequency of cells harboring integrated HIV DNA in sorted CD4^+^ T cell subsets from the same individuals. Undetectable measurements are represented as unfilled symbols, and the half of the LOD are plotted. (**D**) Contribution of each CD4^+^ T cell subset to the pool of HIV-infected cells from each individual. For panels **A**–**D**, each sample is represented by a unique color-coded symbol and unique ID, followed by the number of years on suppressive ART from the first (T1) and second (T2) time point. Data were log_10_-transformed, and Wilcoxon’s matched pairs signed-rank test was used to compare both time points. (**E**) Comparison of the contribution of CD4^+^ T cell subsets to the pool of circulating CD4^+^ T cells and to the pool of HIV-infected cells, summarized by pie charts displaying the mean proportions across individuals at T1 and T2. Wilcoxon’s matched pairs signed-rank test was used to compare the contribution of each CD4^+^ T cell subset to the pool of circulating cells with its contribution to the pool of HIV-infected cells.

To determine whether the inducibility of HIV proviruses may increase as children age, we measured total and integrated HIV DNA, as well as the inducible reservoir by TILDA and the translation-competent reservoir by HIV-Flow, in samples from 8 early-treated, ART-suppressed children collected 2 years apart (1.9 [1.9–2.1] years, Supplemental Table 5). There was a statistically significant decrease in the frequency of cells harboring total HIV DNA over time (ratio = 1.2 [2.9–12.6], *P* = 0.04, Figure 3A). Although they tended to decrease, the frequencies of cells harboring integrated HIV DNA and inducible HIV transcripts (TILDA) did not significantly differ between the 2 time points. Translation-competent reservoirs measured by HIV-Flow were undetectable in all but 1 sample, indicating that the proviral genomes persisting in early-treated children on ART remain poorly inducible during the first 5 years of life.

The relative proportions of CD4^+^ T cell subsets undergo dynamic changes and rapid maturation in the early years of childhood. To determine if the cellular location of HIV reservoirs may be affected by these changes, we measured HIV DNA levels in cell-sorted subsets from the longitudinal samples described above. As expected, the frequency of naive CD4^+^ T cells significantly decreased with age (fold-change = 0.95 [0.92–0.98], *P* = 0.04), while the frequencies of EM subset slightly increased (fold-change = 1.6 [1.3–2.3], *P* = 0.02, Figure 3B). Integrated HIV DNA levels decreased significantly in naive and CM (*P* = 0.008 in both cases, Figure 3C), while the trends observed in TM and EM did not reach statistical significance. The relative contribution of each subset to the pool of cells harboring integrated HIV DNA did not significantly change over time, with CM carrying the bulk of viral genomes at both time points (Figure 3, D and E). In these children, memory cells represented only a mean of 11.8% (SD 4.7) at T1 to 13.6% (SD 4.4) at T2 of all CD4^+^ T cells but harbored 77.3% (SD 22.1) at T1 to 84.5% (SD 15.8) at T2 of the HIV genomes (Figure 3E). Overall, our results indicate that the HIV reservoir in early-treated children remains poorly inducible and primarily located in memory CD4^+^ T cells during the first 5 years of life.

### Long-term HIV reservoir dynamics.

**Figure 4:**
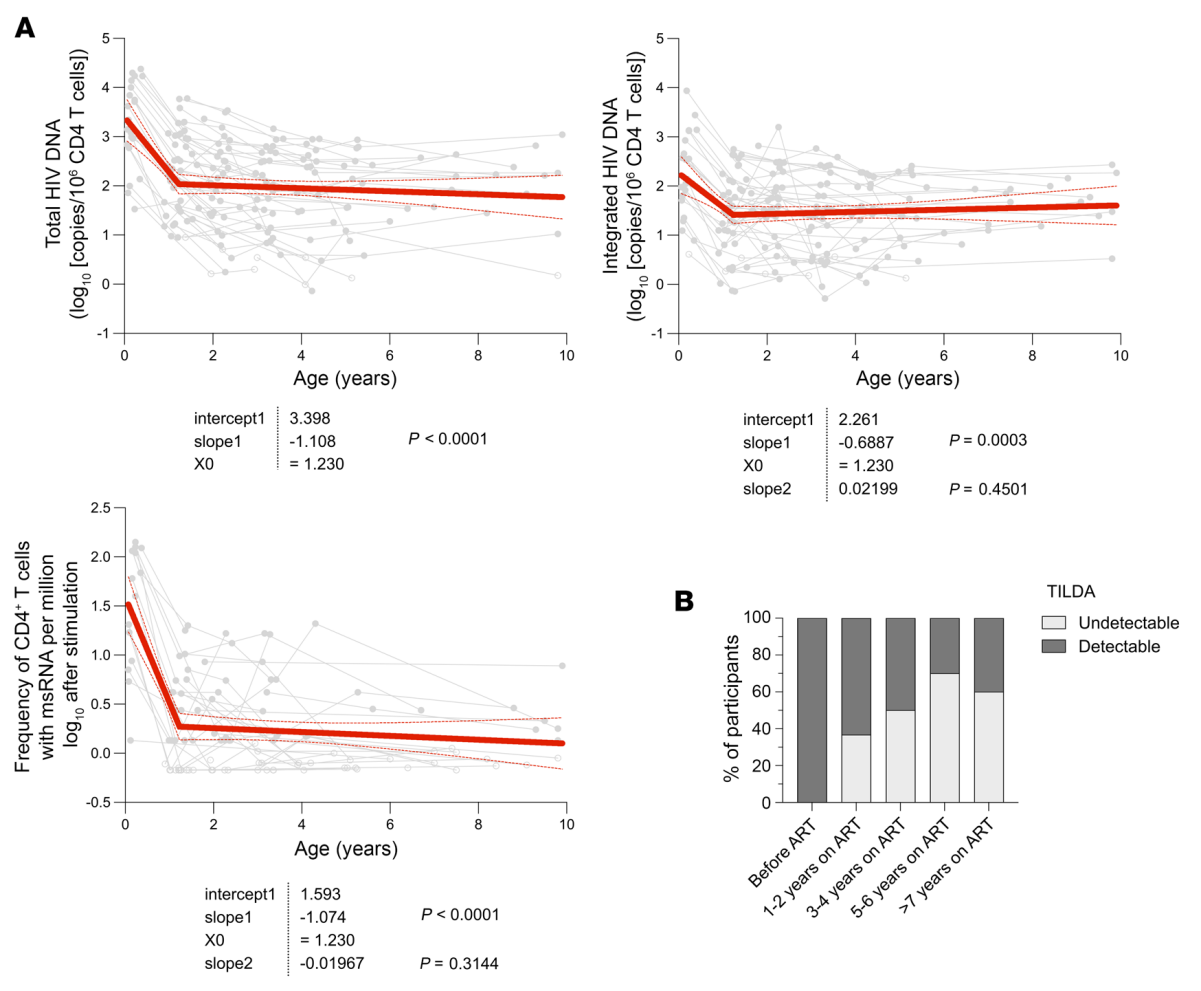
Evolution of the HIV persistence markers over time in early-ART-treated Thai children. (**A**) Long-term dynamics of HIV persistence markers before and after early ART initiation (up to 10 years of follow-up) in 49 vertically infected Thai children. The frequency of cells harboring total (left panel) and integrated (middle panel) HIV DNA in CD4^+^ T cells was measured using real-time PCR, and the frequency of CD4^+^ T cells producing msRNA following 12-hour stimulation with PMA/ionomycin (right panel) was quantified by TILDA. Segmental-linear models were used to measure longitudinal decays in each HIV reservoir marker. Each dot represents a time point analyzed for a given participant, and samples from the same individual are connected. The best fitted model for each virological marker is presented in red. Intercept, as well as slopes (first and second phase decays) are indicated. *P* values indicate whether the slopes are significantly different from 0, indicating a decay in the HIV reservoir markers over time. (**B**) Proportion of samples with detectable and undetectable TILDA values at different time points.

The results of the experiments described above indicate that CD4^+^ T cells harboring inducible HIV genomes are extremely rare after 2–5 years of therapy. To better understand the dynamic of the viral reservoir from ART initiation (i.e., <21 weeks of age) through to 9.9 years of therapy, we used longitudinal samples from 49 children with early-treated perinatal infection (Supplemental Table 6). Total HIV DNA, integrated HIV DNA, and frequency of CD4^+^ T cells producing multiply spliced RNA (msRNA) were detected in all children at high levels before ART initiation (2,189 [691–10,011], 139 [51–845], and 50 [1–114] infected cells per 10^6^ CD4^+^ T cells, respectively, Figure 4A). Longitudinal analysis revealed a biphasic decay of all HIV persistence markers with ART administration, with a rapid initial decline followed by a slower decay. During the first-phase decay (0 to 1.2 years of ART), total HIV DNA, integrated HIV DNA, and TILDA measures rapidly decreased with similar half-lives (decay of –1.11, –0.69, and –1.07 log_10_ infected cells/yr, respectively, *P* < 0.001 in all cases). This was followed by a second phase (after 1.2 years of ART), during which all 3 HIV reservoir markers remained stable (–0.03, 0.02, and –0.02 log_10_ infected cells/yr, respectively, decay *P* = NS). Despite no apparent decrease in the size of the inducible reservoir, the proportion of participants with detectable TILDAs decreased with time on ART: msRNA^+^ cells were detected by TILDA in all children at baseline (100%, *n* = 14), in 59% after 1 to 4 years on ART (*n* = 75), and in less than 40% of the participants after 5 years of therapy (*n* = 25) (Figure 4B). Together, our longitudinal analysis revealed that early-treated children experienced a rapid decline of all HIV markers during the first year of life. During the second phase, HIV DNA remained stable for up to 9.9 years of follow-up, while cells harboring inducible HIV genomes became undetectable at our sampling depth in most participants after 5 years of continuous ART.

### Clonal expansion contributes to the stability of HIV DNA levels.

**Figure 5:**
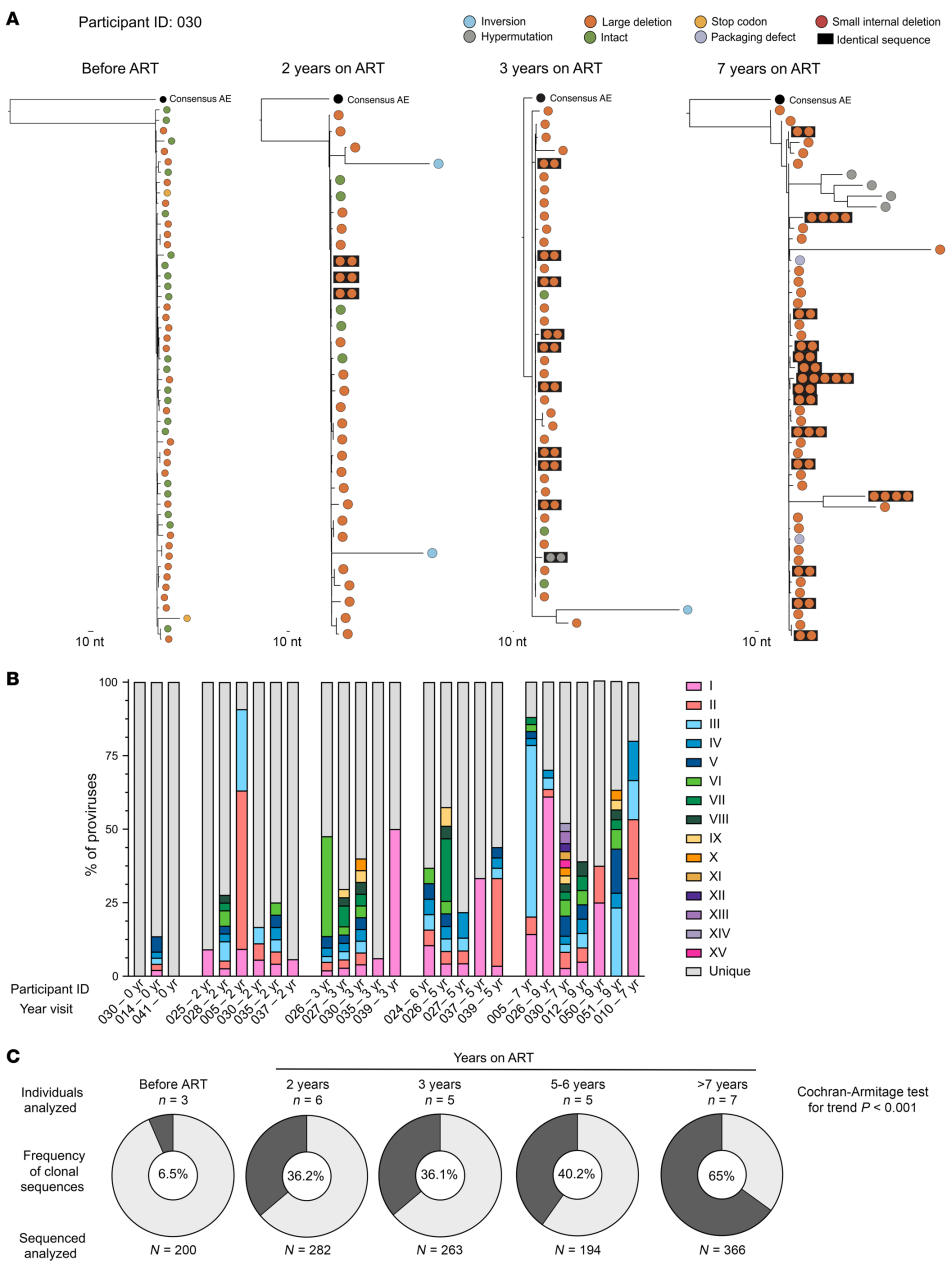
Clonality of the HIV reservoir over time in early-ART-treated Thai children. (**A**) Phylogenetic trees of near full-length HIV genomes of participant 030 at 4 time points (before ART, and after 2, 3, and 7 years on ART). Clonal expansions of 100% identical proviral sequences are clustered together in a dark box. (**B**) Percentage of the HIV reservoir composed of unique proviral sequences (light gray) and of clonal expansions (colors) of 26 samples. Each sample was composed of 1 to 15 clones. (**C**) Pie charts representing the proportion of the HIV reservoir that is composed of clonal HIV genomes (dark gray) or unique ones (light gray) for all samples of each time point. The total number of proviruses per time point is indicated in the center of the pie chart, and the number of samples per time point is noted at the top. *P value* (Cochran-Armitage Test for Trend) indicates that the trend of an increasing proportion of clonal HIV sequences is significant (*P <* 0.001).

The stability of HIV DNA levels in these early-treated children contrasts with the continuous decline reported in adults who received ART in the first few weeks of infection (37). Since the pool of cells harboring HIV DNA is primarily maintained by clonal expansion in adults on ART (38–40), we sought to measure the level of clonality in the pediatric reservoir. We obtained 1,305 near full-length HIV genome sequences from 26 samples (16 participants; from 1 to 4 time points per participant ranging from the day of ART initiation to more than 7 years on ART, Supplemental Table 7). Our sequencing approach covers 92.8% (position 639 to 9,598 on the Consensus AE genome reference) of the whole HIV genome (41) and can be used to document clonal expansion, defined as clusters of 100% identical viral genomes (42). We performed an in-depth analysis of the reservoir clonality in 4 longitudinal samples from participant 30 (Figure 5A). All 52 genomes retrieved from the pre-ART time point were genetically unique, indicating that clonal proliferation is rare during viremia. Small clonal expansions were detected after 2 years of ART, and clonality gradually increased with time on therapy (Figure 5A). Three clusters of proviruses were also shared between 2 time points (data not shown). We used cross-sectional samples of early-treated children followed for up to 9 years to confirm these observations. Clonally expanded proviruses were detected in all samples from children on ART (between 1 and 15 clones per sample) and in only 1 of the 3 viremic samples tested (Figure 5B). Overall, only 6.5% of the viral sequences obtained before ART were clonally expanded. Clonality gradually increased with time on ART from 36% after 2–3 years to 40% after 5–6 years and 65% after more than 7 years of continuous viral suppression (Figure 5C and Supplemental Figure 5A). This increased clonality of HIV genomes during long-term ART was statistically significant (Cochran-Armitage Test: *P* < 0.001) and indicates that clonal expansion is a major contributor to the stability of the pool of cells harboring HIV DNA in early-treated vertically infected children.

### Scarcity of intact HIV genomes.

**Figure 6:**
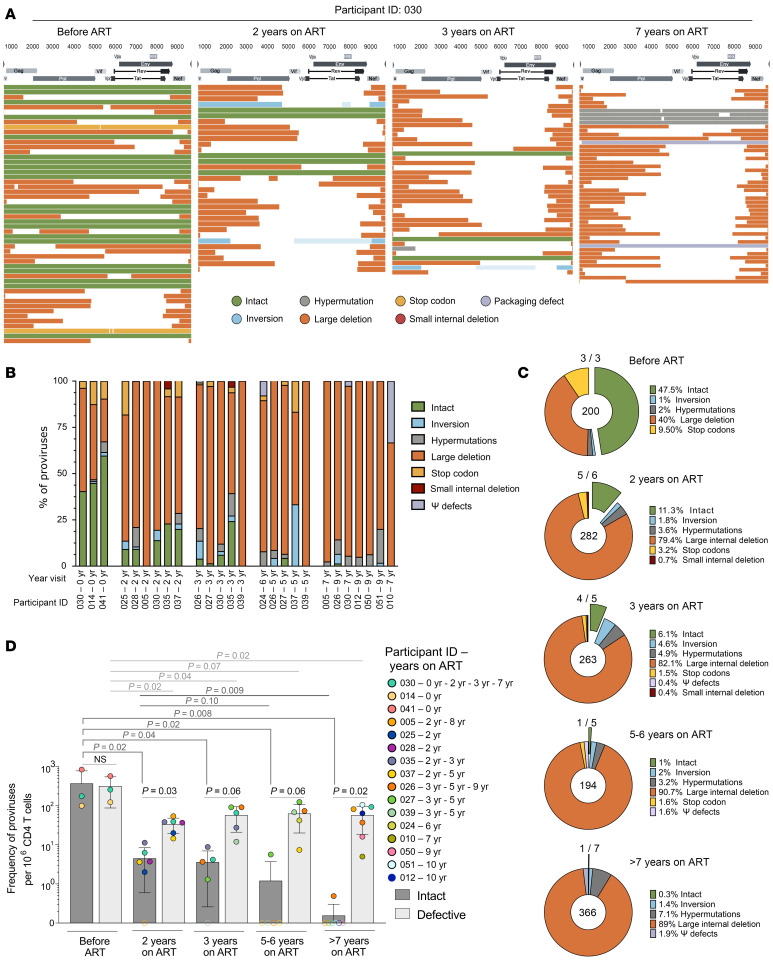
Integrity of the HIV reservoir over time in early-ART-treated Thai children. (**A**) Near full-length HIV genome alignments of participant 030 at 4 time points (before ART, and after 2, 3, and 7 years on ART). Each sequence is color-coded based on its integrity. (**B**) Percentage of each integrity categories of the HIV reservoir of 26 samples. Each sample was composed of 1 to 15 clones. (**C**) Pie charts representing the proportion of each integrity categories of the HIV reservoir for all samples of each time point. The percentage of each category is shown in the legend of the pie chart. The total number of proviruses per time point is indicated in the center of the pie chart, and the number of samples per time point with intact proviruses is noted at the top. *P value* (Cochran-Armitage Test for Trend) indicates that the trend of a decreasing proportion of intact HIV sequences is significant (*P* < 0.001). (**D**) Frequency of defective (light gray bars) and intact (dark gray bars) proviruses sequenced per million CD4^+^ T cells tested. Each dot represents the value for a specific sample, and each PID is color-coded. Bar graph shows the mean value for each group. Unfilled circle corresponds to undetectable values.

The above results suggest that the pool of cells harboring HIV genomes is maintained by clonal proliferation. The observed decline over time in the frequency of cells containing inducible HIV genomes suggests that genetically intact HIV is gradually and selectively depleted during long-term ART. To test this hypothesis, we interrogated the genetic integrity of the proviral reservoir over time by sequencing 1,305 near full-length HIV genomes from 26 samples from children with perinatal HIV, from ART initiation and after up to 9.9 years on ART. We used 4 longitudinal samples from participant 30 to perform an in-depth analysis of the reservoir integrity (Figure 6A). Before ART initiation, 40% (21/52) of the HIV genomes were intact. The proportion of genetically intact proviral sequences rapidly decreased after ART initiation, from 14% after 2 years (5/36) to 6% after 3 years (3/50). Remarkably, none of the 73 proviral sequences obtained from a sample collected after 7 years of ART were predicted to be intact (Figure 6A). Similar observations were made when we analyzed proviral sequences in multiple participants collected at several time points: intact proviruses were detected in samples from all 3 participants sampled before ART initiation, while the proportion of children carrying genetically intact genomes decreased with time on ART (5/6 after 2 years, 4/5 after 3 years, 1/5 after 5–6 years, and 1/7 after more than 7 years on ART, Figure 6B). Overall, genetically intact genomes represented 47.5% of all proviruses before ART (95/200), 11.3% (32/282) after 2 years, 6.1% (16/263) after 3 years, 1.0% (2/194) after 5–6 years, and 0.3% (1/366) after more than 7 years on ART (Figure 6C and Supplemental Figure 5B). Large internal deletions were the most prominent types of defects at all time points, and their proportion tended to gradually increase with time.

We also calculated the frequencies of CD4^+^ T cells harboring intact and defective proviruses in these early-treated Thai children. Before ART initiation, CD4^+^ T cells harboring intact and defective proviruses were detected at similar frequencies (371 [100–842] intact and 317 [123–570] defective proviruses per million CD4^+^ T cells). Frequencies of cells with defective proviruses rapidly decreased during the first 2 years of ART (33.7 cells per million CD4^+^ T cells, *P* = 0.02) and remained stable afterward (57.5 after 3 years, 63.1 after 5–6 years, 57.4 after more than 7 years) (Figure 6D). In sharp contrast, frequencies of cells harboring intact HIV genomes continuously decreased during years of viral suppression (4.5, 3.6, 1.1, and 0.07 cells per million CD4^+^ T cells after 2, 3, 5–6, and >7 years of ART, respectively). Overall, while the pool of cells harboring HIV DNA was stable, genetically intact HIV genomes were selectively depleted and exceedingly rare after 7 years of therapy, indicating that children who initiated ART early after perinatal infection have an exquisite capacity to naturally clear their viral reservoir.

## Discussion

In this study, we analyzed the inducibility and genetic intactness of the HIV reservoir in Thai children who initiated ART during the first weeks of life. By combining the measurements of frequencies of cells harboring HIV genomes and those producing transcripts, proteins, or infectious virions together with proviral sequencing of near full-length genomes, our results reveal insights into the dynamics of the HIV reservoir in this pediatric population.

First, our results indicate that while HIV genomes were detectable in most of the samples we analyzed, only a small fraction produced msRNA (~3%), the viral protein Gag (<1%), and infectious virions (<0.3%) upon stimulation. These proportions appear to be 2 to 5 times lower than those measured in adults on ART (43), suggesting that intrinsic differences exist between the adult and pediatric reservoirs. One possibility is that intact HIV proviruses in children preferentially reside in cells with reduced responsiveness to stimulation. Alternatively, the pediatric immune system, particularly in the context of early ART, may more effectively suppress viral transcription or eliminate cells with inducible proviruses.

We first explored the possibility that this restricted inducibility may be related to the cellular location of the reservoir. Our analysis of sorted T cell subsets from children on ART showed that memory CD4^+^ T cells harbored the majority of integrated HIV DNA, as observed in adults. This is not surprising given that CCR5, the major coreceptor used by HIV during transmission, including in the context of perinatal infection (44, 45), is barely expressed by naive cells, whereas all memory cells and particularly the most differentiated ones (EM) readily express this receptor (46). As such, we observed that while naive cells represented 83% of all CD4^+^ T cells, their contribution to the pool of cells harboring HIV DNA was only 17%, which is in line with the results reported by Katusiime et al (24). Interestingly, in the few participants we followed longitudinally, this contribution decreased (from 23% to 16% after 2 additional years of ART), while the contribution of the long-lived CM cells remained stable. This is in line with the relatively short longevity of naive cells compared with memory cells and indicates that, as observed in adults, HIV preferentially seeds long-lived memory subsets, which are well suited to sustain the reservoir over extended periods (33). Among the memory subsets, we found that CM cells harbored more than 50% of all integrated proviruses. This is again similar to what was previously reported in adults treated in chronic infection (33), whereas this proportion appears relatively high when compared with adults treated during acute infection in whom CM cells are relatively protected (47). This difference may reflect the distinct composition of memory subsets in these 2 groups: CM cells account for more than 80% of memory CD4^+^ T cells in neonates with HIV, whereas they represent only 30% of the memory pool in adults with acute infection. Given that CM cells are long-lived and capable of self-renewal, it is plausible that HIV-infected CM cells contribute to an extremely stable reservoir in children. Supporting our hypothesis, our longitudinal measurements of HIV DNA levels revealed that, following a fast initial decay during the first year of ART, the frequency of cells harboring integrated HIV DNA remained remarkably stable and even tended to increase in some children, in line with a recent study in which HIV DNA levels were measured in Kenyan children with HIV who initiated ART between 1 and 12 months of age (48). The analysis of near full-length genome sequences in our study revealed a likely explanation for this phenomenon: The clonality of the HIV reservoir (defined as the proportion of identical genomes retrieved in multiple copies) rapidly increased after the first year of ART, indicating that the proliferation of memory CD4^+^ T cells is a major contributor to the maintenance of HIV DNA levels in early-treated Thai children. While previous studies have reported large clones of cells harboring solo long terminal repeats (LTRs), in children on ART (49), our findings indicate that longer proviruses can also undergo clonal expansion and contribute significantly to reservoir persistence.

Our longitudinal analysis revealed that the exquisite stability of the pool of cells harboring integrated HIV DNA contrasted sharply with other measures of the reservoir. Indeed, when we measured the frequency of cells that produced multiply spliced HIV RNA upon stimulation using TILDA, we observed a continuous decay in this marker, with most of the samples collected from children on ART for more than 7 years being negative by this relatively sensitive assay. Similarly, nearly all samples tested for inducible p24 expression using the HIV-Flow assay or for replication-competent virus with the mQVOA failed to yield a measurable signal. Here again, the sequencing of near full-length proviruses provided an explanation for these intriguing findings. By sequencing a total of 1,305 HIV proviruses, we observed a marked, selective decay of genetically intact HIV genomes. Remarkably, only a single intact provirus was retrieved from samples from 7 children who had been on ART for more than 7 years, whereas the frequency of cells harboring defective genomes, particularly those with large internal deletion, remained relatively stable. Together, these results support a model in which all memory CD4^+^ T cells undergo proliferation and that this process differentially affects cells harboring intact versus defective HIV genomes: while defective genomes can be duplicated through cell division without triggering cytopathic effects or immune clearance, cells with genetically intact HIV genomes may be preferentially eliminated. Interestingly, results from a recent study by Hasson et al. (23) suggested that different selection pressures shape the proviral landscape in children compared with adults. In line with this, Bone et al. (27) reported that a functional NK cell signature is associated with a smaller reservoir, suggesting that innate immune responses may play a role in that process in young adults who acquired HIV perinatally. Since it was recently demonstrated that CD4^+^ T cells with an intact provirus have a profound proliferative defect in response to TCR stimulation (50), we cannot exclude the possibility that CD4^+^ T cells carrying intact genomes did not undergo proliferation. Therefore, the selective advantage of cells with defective proviruses to generate large clones could be attributed to their capacity to escape immune responses, to their superior proliferative potential, or both. Notably, 1 child (ID 005, 2 years on ART) showed large expansions (3 clones comprising 90% of sequences), together with a relatively large frequency of cells harboring HIV DNA but a negative TILDA, consistent with the preferential proliferation of cells carrying defective, noninducible proviruses.

These results raise important implications for cure strategies in children with perinatally acquired HIV. The profound decay of genetically intact and inducible HIV genomes in early-treated children suggests that this population may be particularly well positioned to benefit from interventions aimed at achieving HIV remission. Importantly, a few cases of posttreatment control have already been reported in children, demonstrating that durable viral suppression after ART interruption is possible in this population (51–53). The viral profiles of vertically infected children on long-term ART in our study mirror those observed in adult posttreatment controllers and support the idea that, in some cases, the reservoir may decay to a point at which treatment interruption could be safely considered. Although structured analytical treatment interruptions in carefully selected pediatric participants could help define the thresholds of viral control, those should be considered after demonstrating a failure to detect infected cells with a denominator in the billions.

Our findings also emphasize the limitations of total HIV DNA as a biomarker of the replication-competent reservoir, particularly in children. Despite stable HIV DNA levels, we observed a marked reduction in the intact and inducible reservoirs over time. Therefore, quantification of total or even integrated DNA alone may not accurately reflect the true reservoir burden, and functional and genetic assessments should be incorporated into reservoir monitoring — especially in the context of cure trials.

There are limitations to our study. The number of participants with longitudinal measurements of inducibility, proviral sequencing, and cell subset sorting was limited, largely due to restricted sample availability — a common challenge in pediatric cohorts. Assays such as mQVOA and HIV-Flow require large numbers of cells, which constrained the number of participants. The relatively low cell input in the assays reduced the sensitivity for detecting cells producing viral proteins and replication-competent HIV. In addition, our analyses were limited to peripheral blood, which may not fully capture the reservoir harbored in tissue compartments. Finally, we did not sort T memory stem cells (TSCM) separately; grouping them with naive cells may dilute naive estimates and obscure a reservoir-enriched TSCM subset. Despite these limitations, this study represents, to our knowledge, the most comprehensive longitudinal analysis to date of both inducible and genetically intact HIV genomes in early-treated perinatally infected children.

In summary, our study provides strong evidence that early ART initiation in perinatally infected children leads to a profound and sustained reshaping of the viral reservoir. While total HIV DNA stabilizes after the first year of therapy, genetically intact and inducible HIV genomes continue to decay, becoming exceedingly rare after 5–7 years of treatment. These findings highlight the potential of early-treated children as a priority population for HIV remission strategies and underscore the need to incorporate sensitive and specific inducibility assays in future pediatric HIV cure research.

## Methods

### Sex as a biological variable.

This study evaluated samples from male and female children who acquired HIV perinatally. Similar findings are reported for both sexes.

### Study design.

The overall objective of this study was to measure the size and characterize the pool of HIV-infected cells in blood of vertically infected early-treated ART children (*N* = 62). For that, we used samples from different cohorts: RV475/HIVNAT209, HIVNAT209-ext, and RV474/HIVNAT194. Due to sample limitations, inherent to working with pediatric populations, where blood volumes must be minimized, different samples from the children were used to address specific research questions, as specified in the figures and corresponding tables.

Thai children vertically infected with HIV who initiated ART within the first 6 months of life were enrolled in the RV475/HIVNAT209 study (*N* = 58) and followed longitudinally for up to 6 years. Of these, 17 children continued in the HIVNAT209ext extension study, which extended follow-up to 10 years of age. Additionally, we included samples from 4 participants in the RV474/HIVNAT194 study, who initiated ART during the first 6 months of life and maintained sustained suppressed plasma viremia (<400 HIV RNA copies/mL).

These studies were conducted at 8 sites in Thailand: the HIV Netherlands Australia Thailand Research Collaboration (King Chulalongkorn Memorial Hospital), Siriraj Hospital, HatYai Hospital, Srinagarind Hospital, Chiangrai Prachanukroh Hospital, Nakornping Hospital, Prachomklao Hospital, and Queen Sirikit National Institute of Child Health. Caregivers provided consent.

### Total and integrated HIV DNA from enriched CD4^+^ T cells.

Total CD4^+^ T cells were isolated by negative magnetic selection (EasySep Human CD4^+^ T Cell Enrichment Kit, STEMCELL Technologies) from frozen PBMCs. Purity was assessed by flow cytometry, using the following panel: CD3-AF700, CD4-APC, CD8-PerCP-cy5.5, CD14 V450, and CD69-PE-Cy7 (Supplemental Table 8). Purity was >98% in all cases. Pellets of enriched CD4^+^ T cells (0.5 × 10^6^ to 1 × 10^6^ CD4^+^ T cells) were digested with proteinase K lysis buffer (0.1 M Tris HCl, 0.5 M KCl, 10 mg/mL proteinase K from Life Technologies 25530-015) to measure total (5′-LTR-gag) and integrated (3′-LTR-alu) HIV DNA by real-time PCR as previously described (36) (Supplemental Figure 1A). Samples for which <50,000 cells were analyzed were excluded from the analysis.

### Frequency of CD4^+^ T cells with multiply spliced HIV RNA (TILDA).

TILDA was performed as previously described (54) with minor modifications to the original protocol. The detailed procedure is described in the Supplemental Methods.

### HIV-Flow and cell sorting.

Between 10 × 10^6^ and 50 × 10^6^ PBMCs were thawed and enriched for CD4^+^ T cells to perform HIV-Flow assay (43) (Supplemental Figure 1A). Briefly, after 1 hour of preincubation with 5 μg/mL Brefeldin A (BFA) and 24 hours of stimulation with of 162 nM PMA and 1 μg/mL ionomycin in the presence of ARTs (200 nM lamivudine and 200 nM raltegravir), extracellular staining was performed using the following antibodies: CD3-AF700, CD4-FITC, CD45RA-BV786, CD27-BV421, CCR7-BB700, and live/dead aqua vivid (Thermo Fisher Scientific, Supplemental Table 8). Cells were simultaneously fixed and permeabilized with the FoxP3 Buffer Set (eBioscience, 00-5523-00), followed by intracellular staining of HIV p24 with clone 28B7-APC and clone KC57-PE (Supplemental Table 8). All samples were resuspended at a final concentration of 1 × 10^6^ cells/mL in PBS and filtered prior to cell sorting. In parallel, CD4^+^ T cell subsets were also sorted from unstimulated samples to enable downstream analyses under basal conditions.

Viable CD4^+^ T cells were sorted into naive (CD45RA^+^CCR7^+^CD27^+^), central memory (CM, CD45RA^–^CCR7^+^CD27^+^), transitional memory (TM, CD45RA^–^CCR7^–^CD27^+^), and effector memory (EM, CD45RA^–^CCR7^–^CD27^–^) using a BD FACSAria III sorter. The frequency of p24 double-positive cells (KC57^+^28B7^+^) was determined simultaneously in gated viable CD4^+^ T cells and CD4^+^ T cell subsets. Due to the downregulation of CD27 upon PMA/ionomycin stimulation, naive cells were analyzed as the combined population CD45RA^+^CCR7^+^CD27^+^ (N) and CD45RA^+^CCR7^+^CD27^–^ (Z). In all experiments, CD4^+^ T cells from an HIV-negative control were included to set the threshold of positivity for p24 signals. In addition, samples from adults with HIV treated with ART were used as positive control (Supplemental Figure 1B).

CD4^+^ T cell subsets were sorted and digested with proteinase K lysis buffer to measure integrated (3′-LTR-alu) HIV DNA by real-time PCR as previously described (36). Samples for which <10,000 cells were analyzed were excluded from the analysis.

To assess whether stimulation affected the distribution of CD4^+^ T cell subsets, we performed parallel staining of total PBMCs without stimulation in a subset of available samples (*n* = 11) and compared them with stimulated samples (Supplementary Methods and Supplemental Figure 4B). In parallel, CD4^+^ T cell subsets were sorted from unstimulated samples (*n* = 6) processed using the same staining panel but without PMA/ionomycin stimulation, BFA treatment, or intracellular p24 staining. Integrated HIV DNA measures in sorted CD4^+^ T cell subsets were compared between stimulated and unstimulated conditions (Supplemental Figure 4C).

### mQVOA.

We adapted the mQVOA previously described (55). Briefly, enriched CD4^+^ T cells were serially diluted in Costar plates coated with anti-CD3 (2.5 μg/mL, clone OKT3, Biolegend) and anti-CD28 (1 μg/mL, clone CD28.2, BioLegend 302902) monoclonal antibodies. Five serial 3-fold dilutions were performed at a starting concentration of 1 × 10^6^ cells/well (first dilution in a 24-well plate and following dilutions in a 96-well plate), with 6 replicates per dilution when enough cells were available. After 2 days of stimulation, 50,000 or 10,000 MOLT-4/CCR5^+^ cells (NIH AIDS Reagent Program, 4984) were added to cell culture 24 or 96 well plate cell culture, respectively (day 0). Cell cultures were split twice weekly, and half of cell culture supernatants (500 μL or 100 μL) were collected at days 7, 14, and 21 for quantification of soluble HIV-p24 protein. Supernatants were lysed and kept at –80°C until use. p24 protein was quantified by ELISA as previously described (56). The number of wells positive for soluble p24 protein was determined, and the maximum likelihood method was applied to determine infectious units per million of cells (http://silicianolab.johnshopkins.edu/) (57) (Supplemental Figure 1A).

### Single-amplicon near full-length nested PCR amplification.

Near full-length amplification of HIV genomes was performed as previously described (41, 58, 59) and adapted to the AE clade. Enriched CD4^+^ T cell lysates were first diluted to reach a concentration of 0.5–1 copies of integrated HIV DNA per well, and 12 PCRs of each dilution were performed to determine the proper dilution to achieve 33% of positive wells. HIV proviruses were preamplified in a 25-cycle 3-step PCR in a total volume of 40 μL containing Invitrogen Platinum SuperFi II MasterMix (Thermo Fisher Scientific catalog 12358050) with 0.2 μM of each primer (263-AE F: 5′-AGGGACTCGAAAGCGRAAGT-3′; BLOuter R: 5′-TGAGGGATCTCTAGTTACCAGAGTC-3′). In a second round of amplification 5 μL of each 4× diluted preamplification product was used, using inner primers (652-AE F: 5′-ACTCGAAAGCGRAAGTTCCAGAG-3′; 280-AE R: 5′-CTAGTTACCAGAGTCCTAACACAGAYG-3′) for 30 cycles in a 30 μL final volume reaction. pAE plasmid was used as positive control for PCR amplification. Positive reactions were determined by visualization of amplicons on a 0.8% agarose gel. All amplicons were sequenced using the PacBio next-generation sequencing platform. Each HIV preamplified DNA was reamplified with barcoded inner PCR primers (96 PacBio barcodes). Barcoded amplicons were purified using AMPure XP beads (Beckman Coulter A63881), following manufacturer’s instructions, prior to NanoDrop quantification (Thermo Fisher Scientific). 50 ng of each of the 96-barcoded amplicons were pooled together and sequenced on a Sequel or Sequel II instrument (DNA Link, South Korea).

### Analysis of proviral integrity and clonality.

The analyses used to determine the genetic integrity of the proviral sequences as well as their clonality are described in detail in the Supplemental Methods (41, 58, 59).

### Statistics.

Continuous variables were described using medians and the IQR or mean (SD), whereas categorical factors were reported as percentages. All reservoir data were log_10_-transformed to better meet the assumption of normality. For calculations and representation, we used half the LOD, calculated based on the total number of cells assayed from each sample. Outliers were assessed using the ROUT method (*Q* = 1% and *Q* = 10%); no outliers were detected in any CD4^+^ T cell subset dataset. To calculate the relative contribution of a given subset to integrated HIV DNA, measurements in which integrated HIV DNA was not detected were assigned a value of 0. For paired data, quantitative variables were analyzed using the Wilcoxon’s matched pairs signed-rank test for 2-variable comparisons and a mixed effects model for multiple measurements, followed by Tukey’s multiple-comparison test. For nonpaired data, the Mann-Whitney *U* test and Kruskal-Wallis test were used, with Dunn’s post hoc test to identify differences between the groups. Bivariate associations between continuous variables were assessed by Spearman’s rank correlation coefficient.

Relative proportions of total HIV DNA and its subcomponents (integrated HIV DNA, TILDA, HIV-Flow, and mQVOA measures) were visualized using hierarchical circle packing diagrams. These visualizations were generated using R (version R4.4.0) with the ggplot2 (version 3.5.1) and ggforce (version 0.4.2) packages. The area of each circle was scaled proportionally to the square root of its corresponding measurement value to accurately represent relative magnitudes.

The Cochran-Armitage Test for Trend was used to analyze the distribution of clonal and unique sequences across time points, as well as the integrity analysis. The test was performed using MedCalc Software Ltd. (Version 23.0.9) (https://www.medcalc.org/calc/chisquared-2way.php). Statistical analyses were performed with Prism 10.3.1 (GraphPad Software); significance was determined when *P* ≤ 0.05.

### Study approval.

All studies were approved by the Thai Ministry of Public Health, Nonthaburi, Thailand, and all participating ethical committees, as well as by the Institutional Review Board of the Centre Hospitalier de l’Université de Montréal (2016-6444, CE 15.328 and 2016-6447, CE 15.331; Montréal, Quebec, Canada). The investigators have adhered to the policies for protection of human research participants as prescribed in AR 70–25. Written informed consent was obtained from parents or legal guardians prior to participation.

### Data availability.

All sequences have been submitted to GenBank and are available under the accession numbers PV267021–PV267309 and PP590794–PP591732. All data are available in the main text or the Supporting Data Values file. Deidentified data will be made available after the completion of the study to researchers with an approved protocol who complete a data use agreement. All inquiries should be sent to the corresponding authors.

## Author contributions

MM, CD, AP, LL and RF were responsible for conducting experiments. MM, CD, AP, AL, CR, LL, JM, and RF were responsible for analyzing data. JA, TJ, SB, RN, P Sawangsinth, MS, P Suntarattiwong, KC, PK, TB, WP and SK were responsible for resources. MM, CD, and AP were responsible for visualization. MM, JA, TP, and NC were responsible for funding acquisition. MM, JA, TJ, MS, LT, TP, and NC were responsible for project administration. JA, MS, LT, TP, and NC were responsible for supervision. MM and NC wrote the original draft. MM, CD, AP, AL, CR, JA, LL, TJ, SB, RN, JM, P Sawangsinth, MS, P Suntarattiwong, KC, PK, TB, WP, SK, LT, RF, TP, and NC were responsible for writing — review and editing.

## Conflict of interest

JA received honoraria from Merck, ViiV Healthcare, Gilead Sciences, AbbVie, and Roche/Genentech for participating in advisory meetings.

## Funding support

This work is the result of NIH funding, in whole or in part, and is subject to the NIH Public Access Policy. Through acceptance of this federal funding, the NIH has been given a right to make the work publicly available in PubMed Central.

- Thailand Science Research and Innovation Fund Chulalongkorn University, HEAF67300088 (TP).
- National Institutes of Health grant R01 AI114236 (JA, TP).
- Canadian Institutes for Health Research grant 364408 (NC).
- Réseau SIDA et maladies infectieuses du Fonds de Recherche du Quebec – Santé, FRQ-S (NC).
- Research Scholar Career Award of the Quebec Health Research Fund, FRQ-S, 253292 (NC).
- Ramon y Cajal from the Spanish Ministry of Science and Innovation and State Research Agency and the European Social Fund “investing in your future”, RYC2020-028934-I/AEI/10.13039/501100011033 (MM).
- Scholarship from the Graduate School, Chulalongkorn University, to commemorate the 72nd anniversary of his Majesty King Bhumibol Adulyadej (RN).

## Supplementary Material

Supplemental data

Supporting data values

## Figures and Tables

**Table 1:**
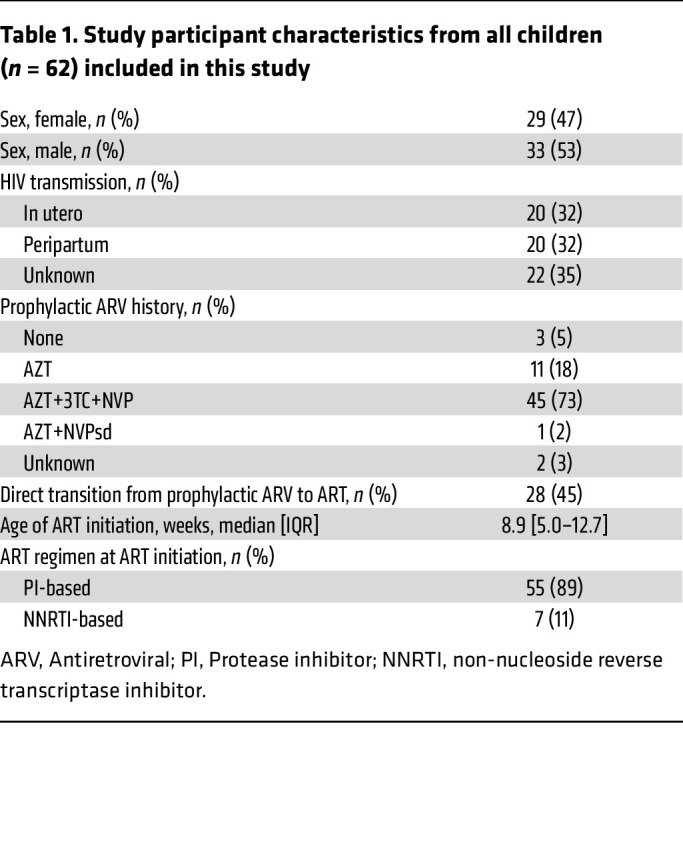
Study participant characteristics from all children (*n* = 62) included in this study

## Version 1 04/02/2026

In-Press Preview

## Version 2 06/01/2026

Electronic publication

## References

[1] Chun TW (1997). Presence of an inducible HIV-1 latent reservoir during highly active antiretroviral therapy. Proc Natl Acad Sci U S A.

[2] Finzi D (1997). Identification of a reservoir for HIV-1 in patients on highly active antiretroviral therapy. Science.

[3] Persaud D (2000). A stable latent reservoir for HIV-1 in resting CD4^+^ T lymphocytes in infected children. J Clin Invest.

[4] Wong JK (1997). Recovery of replication-competent HIV despite prolonged suppression of plasma viremia. Science.

[5] Ajibola G (2021). Viral reservoir in early-treated human immunodeficiency virus-infected children and markers for sustained viral suppression. Clin Infect Dis.

[6] Garcia-Broncano P (2019). Early antiretroviral therapy in neonates with HIV-1 infection restricts viral reservoir size and induces a distinct innate immune profile. Sci Transl Med.

[7] Kuhn L (2018). Age at antiretroviral therapy initiation and cell-associated HIV-1 DNA levels in HIV-1-infected children. PLoS One.

[8] Luzuriaga K (2014). HIV type 1 (HIV-1) proviral reservoirs decay continuously under sustained virologic control in HIV-1-infected children who received early treatment. J Infect Dis.

[9] McManus M (2016). Early combination antiretroviral therapy limits exposure to HIV-1 replication and cell-associated HIV-1 DNA levels in infants. PLoS One.

[10] Millar JR (2021). Early initiation of antiretroviral therapy following in utero hiv infection is associated with low viral reservoirs but other factors determine viral rebound. J Infect Dis.

[11] Payne H (2021). Early ART-initiation and longer ART duration reduces HIV-1 proviral DNA levels in children from the CHER trial. AIDS Res Ther.

[12] Tagarro A (2018). Early and highly suppressive antiretroviral therapy are main factors associated with low viral reservoir in European perinatally HIV-infected children. J Acquir Immune Defic Syndr.

[13] Bitnun A (2014). Early initiation of combination antiretroviral therapy in HIV-1-infected newborns can achieve sustained virologic suppression with low frequency of CD4+ T cells carrying HIV in peripheral blood. Clin Infect Dis.

[14] Ajibola G (2023). Brief report: long-term clinical, immunologic, and virologic outcomes among early-treated children with HIV in Botswana: a nonrandomized controlled clinical trial. J Acquir Immune Defic Syndr.

[15] Massanella M (2021). Continuous prophylactic antiretrovirals/antiretroviral therapy since birth reduces seeding and persistence of the viral reservoir in children vertically infected with human immunodeficiency virus. Clin Infect Dis.

[16] Martínez-Bonet M (2015). Establishment and replenishment of the viral reservoir in perinatally HIV-1-infected children initiating very early antiretroviral therapy. Clin Infect Dis.

[17] Avettand-Fenoel V (2021). Initiating antiretroviral treatment early in infancy has long-term benefits on the human immunodeficiency virus reservoir in late childhood and adolescence. Clin Infect Dis.

[18] Foster C (2021). The CARMA study: early infant antiretroviral therapy-timing impacts on total HIV-1 DNA quantitation 12 years later. J Pediatric Infect Dis Soc.

[19] Eriksson S (2013). Comparative analysis of measures of viral reservoirs in HIV-1 eradication studies. PLoS Pathog.

[20] Ho Y-C (2013). Replication-competent noninduced proviruses in the latent reservoir increase barrier to HIV-1 cure. Cell.

[21] Koofhethile CK (2023). HIV-1 reservoir evolution in infants infected with clade C from Mozambique. Int J Infect Dis.

[22] Rainwater-Lovett K (2017). Paucity of intact non-induced provirus with early, long-term antiretroviral therapy of perinatal HIV infection. PLoS One.

[23] Hasson JM (2025). Differential HIV-1 proviral defects in children vs. Adults on antiretroviral therapy. Viruses.

[24] Katusiime MG (2025). Divergent populations of HIV-infected naive and memory CD4^+^ T cell clones in children on antiretroviral therapy. J Clin Invest.

[25] Hartana CA (2022). Immune correlates of HIV-1 reservoir cell decline in early-treated infants. Cell Rep.

[26] Sun W (2024). Footprints of innate immune activity during HIV-1 reservoir cell evolution in early-treated infection. J Exp Med.

[27] Bone B (2025). Distinct viral reservoirs and immune signatures in individuals on long-term antiretroviral therapy with perinatally acquired HIV-1. Cell Rep Med.

[28] Vela LC (2025). Profound reduction of HIV-1 reservoir cells over 3 decades of antiretroviral therapy started in early infancy. JCI Insight.

[29] van Zyl GU (2015). Early antiretroviral therapy in South African children reduces HIV-1-infected cells and cell-associated HIV-1 RNA in blood mononuclear cells. J Infect Dis.

[30] Katusiime MG (2020). Intact HIV proviruses persist in children seven to nine years after initiation of antiretroviral therapy in the first year of life. J Virol.

[31] Bale MJ (2021). Early emergence and long-term persistence of HIV-infected T-cell clones in children. mBio.

[32] Dhummakupt A (2020). Differences in inducibility of the latent HIV reservoir in perinatal and adult infection. JCI Insight.

[33] Chomont N (2009). HIV reservoir size and persistence are driven by T cell survival and homeostatic proliferation. Nat Med.

[34] Mavigner M (2018). Simian immunodeficiency virus persistence in cellular and anatomic reservoirs in antiretroviral therapy-suppressed Infant Rhesus Macaques. J Virol.

[35] Sleasman JW (1996). CD4+ memory T cells are the predominant population of HIV-1-infected lymphocytes in neonates and children. AIDS.

[36] Vandergeeten C (2014). Cross-clade ultrasensitive PCR-based assays to measure HIV persistence in large-cohort studies. J Virol.

[37] Massanella M (2021). Long-term effects of early antiretroviral initiation on HIV reservoir markers: a longitudinal analysis of the MERLIN clinical study. Lancet Microbe.

[38] Bui JK (2017). Proviruses with identical sequences comprise a large fraction of the replication-competent HIV reservoir. PLoS Pathog.

[39] Wagner TA (2014). HIV latency. Proliferation of cells with HIV integrated into cancer genes contributes to persistent infection. Science.

[40] Maldarelli F (2014). HIV latency. Specific HIV integration sites are linked to clonal expansion and persistence of infected cells. Science.

[41] Dufour C (2023). Phenotypic characterization of single CD4+ T cells harboring genetically intact and inducible HIV genomes. Nat Commun.

[42] Cole B (2021). In-depth single-cell analysis of translation-competent HIV-1 reservoirs identifies cellular sources of plasma viremia. Nat Commun.

[43] Pardons M (2019). Single-cell characterization and quantification of translation-competent viral reservoirs in treated and untreated HIV infection. PLoS Pathog.

[44] Ometto L (2000). Co-receptor usage of HIV-1 primary isolates, viral burden, and CCR5 genotype in mother-to-child HIV-1 transmission. AIDS.

[45] Salvatori F, Scarlatti G (2001). HIV type 1 chemokine receptor usage in mother-to-child transmission. AIDS Res Hum Retroviruses.

[46] Sallusto F (1998). Flexible programs of chemokine receptor expression on human polarized T helper 1 and 2 lymphocytes. J Exp Med.

[47] Leyre L (2020). Abundant HIV-infected cells in blood and tissues are rapidly cleared upon ART initiation during acute HIV infection. Sci Transl Med.

[48] Reeves DB (2025). Intact HIV DNA decays in children with and without complete viral load suppression. PLoS Pathog.

[49] Botha JC (2023). The largest HIV-1-infected T cell clones in children on long-term combination antiretroviral therapy contain solo LTRs. mBio.

[50] Kufera JT (2024). CD4+ T cells with latent HIV-1 have reduced proliferative responses to T cell receptor stimulation. J Exp Med.

[51] Persaud D (2013). Absence of detectable HIV-1 viremia after treatment cessation in an infant. N Engl J Med.

[52] Violari A (2019). A child with perinatal HIV infection and long-term virological control following antiretroviral treatment cessation. Nat Commun.

[53] Frange P (2016). HIV-1 virological remission lasting more than 12 years after interruption of early antiretroviral therapy in a perinatally infected teenager enrolled in the French ANRS EPF-CO10 paediatric cohort: a case report. Lancet HIV.

[54] Procopio FA (2015). A novel assay to measure the magnitude of the inducible viral reservoir in HIV-infected individuals. EBioMedicine.

[55] Massanella M (2018). Improved assays to measure and characterize the inducible HIV reservoir. EBioMedicine.

[56] Bounou S (2002). Presence of host ICAM-1 in laboratory and clinical strains of human immunodeficiency virus type 1 increases virus infectivity and CD4(+)-T-cell depletion in human lymphoid tissue, a major site of replication in vivo. J Virol.

[57] Rosenbloom DIS (2015). Designing and interpreting limiting dilution assays: general principles and applications to the latent reservoir for human immunodeficiency virus-1. Open Forum Infect Dis.

[58] Sannier G (2021). Combined single-cell transcriptional, translational, and genomic profiling reveals HIV-1 reservoir diversity. Cell Rep.

[59] Dufour C (2023). Near full-length HIV sequencing in multiple tissues collected postmortem reveals shared clonal expansions across distinct reservoirs during ART. Cell Rep.

