# Supplementary material for: Rapid and profound decay of inducible and intact HIV genomes in early-treated Thai children
Source: J Clin Invest. 2026 Apr 2;136(11):e198054. doi: 10.1172/JCI198054 (PMC13221237; doi:10.1172/JCI198054)
Supplement: Supplemental data [file jci-136-198054-s072.pdf]

Supplementary material for:

Rapid and profound decay of inducible and intact HIV genomes in early treated Thai children

Marta Massanella, Caroline Dufour, Amélie Pagliuzza, Audrée Lemieux, Corentin Richard, Jintanat Ananworanich, Louise Leyre, Thidarat Jupimai, Supranee Buranapraditkun, Rapisa Nantanee, Julie L. Mitchell, Panadda Sawangsinth, Mark de Souza, Piyarat Suntarattiwong, Kulkanya Chokephaibulkit, Lydie Trautmann, Rémi Fromentin, Thanyawee Puthanakit, Nicolas Chomont; on behalf of the HIVNAT209 and HIVNAT194 study groups

The PDF file includes:

Supplemental Acknowledgments
Supplementary Methods
Tables S1 to S8
Figures S1 to S5

Supplemental Acknowledgments

Research Sites: 1) HIV-NAT, The Thai Red Cross AIDS and Infectious Diseases Research Centre /Center of Excellence in Pediatric Infectious Diseases and Vaccines, Chulalongkorn University: Torsak Bunupuradah, Stephen Kerr, Sasiwimol Ubolyam, Apicha Mahanontharit, Naphassanant Laopraynak, Preeyarach Klaytong, Tulathip Suwanlerk, Thita Pitimahajanaka, Naruporn Kasipong, Thornthan Noppakaorattanamanee, Kesdao Nanthapisal, Umaporn Methanggool, Thatri Iampornsin, Sasithorn Burichai, Yupawadee Jummanee, Sudarat Soongpankeeree, Monta Tawan, Tuangtip Theerawit, Juthamanee Moonwong, Patchareeyawan Srimuen, Chutima Saisaengjan, Wasana Prasitsuebsai, Watsamon Jantarabenjakul, Suvaporn Anugulruengkitt, Wipaporn Natalie Songtaweesin, Jinnaphak Seesuksai, Pattarawat Thantiworasit, Nitikarn Pinyoying. 2) Queen Sirikit National Institute of Child Health: Pugpen Sirikutt, Pimsiri Leowsrisook, Yosawadee Na Nakorn, Naruemon Sassungnune; 3) Siriraj Hospital, Mahidol University: Kanokkarn Wongmayurachat; 4) Srinagarind Hospital, Khon Kaen University: Chanasda Kakkaew, Somjai Rattanamanee; 5) Phrachomklao Hospital: Manee Yentang, Patcha Panyim, Janyarak Punyim; 6) Nakornping Hospital: Thida Namwong, Siripim Kamphaengkham, Supanpilat Chaisri; 7) Chiangrai Prachanukroh Hospital: Rawiwan Hansudewechakul, Areerat Khongponoi; 8) Hat Yai Hospital: Ratchanee Saksawad,Usa Sukhaphan, Arena Laeyuheem. Laboratory and Network: 1) PHPT: Gonzague Jourdain, Nicole Ngo-Giang-Huong; 2) Vaccine and Cellular Immunology laboratory-Chulalongkorn University: Sunee Sirivichayakul; 3) The National Cancer Institute, NIH: Frank Maldarelli; 4) The University of Sydney: Sarah Palmer. MoPH and ACC network: 1) Thailand MOPH-U.S. CDC Collaboration: Michael Martin, Rangsima Lolekha, Thananda Naiwatanakul, Worawan Faikratok, Benjamas Baipluthong; 2) The Bureau of Health Promotion, MoPH: Danai Teewunda, Sarawut Boonsuk, Chaweewan Tonputsa, Pariwat Tangpong; 3) Clinical Research Center, MoPH: Archawin Rojanawiwat, Hansa Thaisri, Wiroi Puangtubtim, Chaidan Boonrossak; 4) The Bureau of AIDS TB and STIs, MoPH: Sumet Ongwandee, Walairat Chaifoo, Cheewanan Lertpiriyasuwat, Patcharaporn Pawapootarnont, Jiraporn Chucherd,; 5) Faculty of Associated Medical Sciences, Chiang Mai University: Tanawan Samleerat; 6) National Health Security Office: Suchada Chaiwut; MHRP: Suteeraporn Pinyakorn, Oratai Butterworth, Madelaine Ouellette, Nelson Michael, Robert Gramzinski.

Supplementary Materials and Methods

Frequency of CD4+ T cells with multiply spliced HIV RNA (TILDA)

Enriched CD4+ T cells were stimulated for 12 h with 100 ng/mL PMA (Sigma, P8139) and 1 µg/mL ionomycin (Sigma, I9657) in the presence of antiretroviral drugs (200 nM raltegravir and 100 nM 3TC). At the end of the stimulation, supernatants were stored at -80°C to assess viral production and cells were immediately serially diluted and distributed at 18,000, 9,000, 3,000 and 1,000 cells per well in a 384 well-plate (up to 24 replicates per dilution, depending on cell availability) containing 5µL of 2x Reaction Mix with Rox from Superscript III Platinum One-step qRT-PCR kit (ThermoFisher) (fig. S1A). CD4+ T cells after stimulation were stained to assess cell-death and activation using the following panel of antibodies: CD3-AF700, CD4-APC, CD8-PerCP-cy5.5, CD14 V450, CD69-PE-Cy7 (table S5) and Aqua vivid Live/dead (Life Technology, L34957). Cell viability and activation were analyzed on a BD LSRIII instrument. Only samples with >50% viable cells and >95% activated cells (as measured by CD69 expression, fig. S1, C and D) were included in the analysis.

Pre-amplification was carried out by adding 5 µL of a PCR mix containing 0.2 µL Superscript III Platinum One-step qRT-PCR (ThermoFisher), 0.1 µL RNase inhibitor (ThermoFisher), 0.125 µL of each primer (at 20 µM), 2.2 µL Tris–EDTA (TE) buffer and 2.25 µL H2O to each well (final reaction volume=11µL). Oligonucleotides sequences were adapted for clade CRF01_AE, which represent ~80% of the circulating HIV in Thailand: Forward: tat1.4-AE–5'-TGGCAGGAAGAAGCGGAAG-3' and Reverse: RevAE–5'-TGTCTCTGYCTTGCTCKCCACC-3'. Pre-amplification was carried out using the following steps: reverse transcription at 50 °C for 15 min, denaturation at 95 °C for 2 min, 24 cycles of amplification (95 °C 15s, 60 °C 4min). 1µL of reverse transcribed and pre-amplified cDNA was transfer to new plate containing in eah well 5µL of Perfecta Fast advance master mix (VWR), 3.3µL H2O, 0.2µL of each primer (at 20µM) and 0.3µL of probe (at 5µM), final reaction volume=10µL. Sequences of primers and probe for tat/rev real-time PCR reaction were also adapted: Forward: tat2AE–5'-GCAGTAAGGATCATCAAAATCCTATACCAGAGCA-3'; Reverse: RevAE; and probe: msHIVAE-FAM/ZEN–5'-TTCYTTCGGGCCTGTCGGGTTCC-3'. Positive wells at each dilution were counted and the maximum likelihood method was used to calculate the frequency of cells with inducible HIV msRNA (http://bioinf.wehi.edu.au/software/elda).

PBMC staining to assess CD4+ T-cell subset distribution

To evaluate whether PMA/ionomycin stimulation affected the distribution of CD4+ T cell subsets, PBMCs from a subset of available samples (n = 11) were stained and analyzed without stimulation. Briefly, PBMCs were thawed and ~0.5×10^6 cells were stained with a live/dead viability dye (Aqua Vivid, Thermo Fisher) together with surface antibodies (Table S8). For the analysis presented here, CD4+ T cell subsets were defined using CD3-AF700, CD4-BUV496, CD45RA-BV786, CD27-APC-eFluor780, and CCR7-PE-CF594. Additional markers included in the full panel were not used for this analysis. After staining, cells were acquired on an LSR-II flow cytometer and analyzed using FlowJo to determine the frequencies of CD4+ T-cell subsets in unstimulated PBMCs. Frequencies were then compared with those obtained after PMA/ionomycin stimulation in the HIV-Flow assay using the same samples.

Analysis of proviral integrity and clonality

PacBio demultiplexing procedure was previously described. The demultiplex barcodes analysis was powered by the Lima PacBio software v2.0.0. High-quality phased consensus sequences representing near full HIV-1 genome sequences with high fidelity and without reconstruction were generated with the LAA PacBio algorithm v2.4.2. Any sequences that did not blast with HIV or that lacked one of the primer sequences were discarded from further analysis. A minimum of 10 reads per sequence were used as a coverage threshold.

Integrity analysis was performed as previously described *(38, 56, 57)*. All proviral sequences were aligned to the reference Consensus AE sequence using Multiple Fast Fourier transform algorithm (MAFFT) with strategy E-INS-i and a scoring matrix of 1PAM/k = 2 (online https://mafft.cbrc.jp/alignment/server/ or with Geneious Prime (v2025.0.3) plugging). For integrity analysis, all sequences containing (in this order) inversions, hypermutations, large internal deletions, stop codons, small internal deletions in a coding region, or defects in the Ψ locus were considered

defective. Inversions were detected manually at the alignment step, since only the reverse complement of inversions can be aligned with the reference. Hypermutations were detected using the online HIV Database QC Tool (https://www.hiv.lanl.gov/content/sequence/QC/index.html). Genomes with large internal deletions were defined as any provirus shorter than 8,800 bp (excluding the primer's regions). Stop codons and small internal deletions (>5% of ORF expected length) were identified using both the HIV Database Gene Cutter online tool (https://www.hiv.lanl.gov/content/sequence/GENE_CUTTER/cutter.html) and Proseq IT (https://psd.cancer.gov/tools/pvs_annot.php). Any defect in the Ψ locus (MSD point mutation; stem loop 2 deletion; packaging signal deletion) were determined using the Proseq IT tool and manually confirmed by looking at the alignment with the reference sequence. The intactness of 9 coding regions (gag, pol, vif, vpr, tat, rev, vpu, nef, env) and 2 regulatory regions (Ψ and RRE) were evaluated by assessing the presence of a start codon (except for pol), and the lack of internal stop codons, frameshift or deletion over 5% of the ORF's length. The analysis used the R script IntactnessandClonality.R (https://github.com/alemi055/IntegrityAlgorithm/tree/main).

Clonal sequences were defined as proviral amplicons 100% identical to each other, and were determined using the HIV Database Elim Dupes online tool (https://www.hiv.lanl.gov/content/sequence/elimdupesv2/elimdupes.html), and confirmed using the Geneious Prime diversity tool. Phylogenic trees were built with IQ-Tree2, using a Maximum-likelihood tree GTR + I + G model, 1,000 bootstraps.

Table S1: Mapping of Participant Samples to Figures

ID participant	Fig1	Fig2	Fig 3	Fig4	Number of Samples in Fig4	Fig5-6	Suppl Fig 1	Suppl Fig 2	Suppl Fig 3	Suppl Fig 4	Suppl Fig 5	Observations
1	4y	4y							4y	4y		
2	3y	3y	3y-5y	1y-2y-3y-4y-5y	5			1y-3y	3y	3y		
3	2y	2y	2y-4y	1y-2y-3y-4y	4				2y	2y		
4	2y	2y							2y	2y		
5	1y	1y	1y-5y	1y-2y-3y-4y-5y-8y	6	2y-8y			1y	1y-3y[$]	2y-8y	[$]1y stimulated; 3y Unstimulated
6	1y	1y	1y-3y	0y-1y-2y-3y-7y	5				1y	1y		
7	1y	1y	1y-3y	0y-1y-2y-3y-5y	5				1y	1y		
8	1y	1y					VF		1y	1y - VF		
9	2y	2y	2y-4y	0y-1y-2y-3y-4y	5			1y-2y	2y	2y-3y[$]		[$]2y stimulated; 3y unstimulated
10	1y	1y	1y-3y	1y-2y-3y-7y	4	7y			1y	1y	7y	
11	2y	2y		1y-2y-8y					2y	2y		
12	4y	4y		1y-2y-3y-4y-10y		10y			4y	4y	10y	
13	3y	3y	3y-5y	0y-1y-2y-3y-4y-5y-8y	7			1y-2y-3y	3y	3y		
14	1y	1y		0y-1y-2y	3	0y			1y	1y	0y	
15	1y			0y-1y	2				1y			
16	1y			0y-1y-2y-3y-4y	5				1y			
17	1y						VF		1y	VF		
18	1y			0y-1y-2y	3				1y			
19	1y			0y-1y-2y	3				1y			
20	1y								1y			
21	2y			0y-1y-2y-3y-4y	5				2y			
22	3y			1y-3y-4y-5y-10y	5				3y			
23	3y			1y-3y	2				3y			
24						6y					6y	
25						2y					2y	
26				0y-1y-2y-3y-4y-5y-9y	7	3y-5y-9y				4y	3y-5y-9y	
27				1y-2y-3y-4y-5y	5	3y-5y					3y-5y	
28				2y-3y-4y	3	2y					2y	
29				0y-1y			VF			VF		
30				0y-1y-2y-3y-4y-7y-8y	7	0y-2y-3y-7y				2y	0y-2y-3y-7y	
33							VF			VF		
35				1y-2y-3y	3	2y-3y				2y	2y-3y	
36							VF			VF		
37				1y-2y-3y-5y		2y-5y					2y-5y	
38				1y-2y	2		VF			VF		
39				1y-3y-4y-5y	4	3y-5y				3y	3y-5y	
40							VF			VF		
41				0y-1y		0y					0y	
42				1y-2y			VF			VF		
44							VF			VF		
45				0y-1y-2y-3y-4y-5y	6							
46				1y-2y								
47				0y-1y-2y-3y-4y-5y-9y	7							
48				1y-2y-3y	3							
49				0y-1y-2y-3y-4y-7y	6							
50				1y-2y-4y-5y-9y		9y					9y	
51				1y-3y-4y-5y-10y	5	10y					10y	
53				1y-2y-3y-4y-8y	5							
54				1y-2y-3y-7y	4							
55				0y-1y								
56				0y-1y								
57				0y-1y-2y	3							
58				1y-2y-3y-4y-10y	5							
59				0y-1y-2y-3y	4							
60				1y-3y-5y	3							
61				0y-1y-2y-3y	4							
62				0y-1y-6y								
63				0y-1y								
64				1y-3y-5y								
65				1y-6y	2							
66							VF			VF		
67							VF			VF		

Table S2: Study participant characteristics included in Figure 1

	Markers of HIV persistence<br>n = 23
Age, years, median [IQR]	1.5 [1.2-2.7]
Gender, Female, n (%)	10 (43)
CD4 counts, cells/µl, median [IQR]	2628 [2003-3343]
CD8 counts, cells/µl, median [IQR]	1671 [1251-2467]
Ratio CD4/CD8, median [IQR]	1.32 [1.09-1.85]
VL <50 HIV RNA copies/ml, n (%)	21 (91)[1]
HIV transmission, n (%)	
In utero	10 (43)
Peripartum	4 (17)
Unknown	9 (39)
Prophylactic ARV history, n (%)	
None	1 (4)
AZT	6 (26)
AZT+3TC+NVP	15 (65)
Unknown	1 (4)
Direct transition from prophylactic ARV to ART, n (%)	10 (43)
Duration prophylactic ARV, days, median [IQR]	29 [28-40]
Time between end of prophylactic ARV and ART initiation, days, median [IQR]	16 [0-54]
Age of ART initiation, weeks, median [IQR]	9.9 [4.7-12.6]
Time on ART, years, median [IQR]	1.4 [1.0-2.6]
ART regimen, n (%)	
PI-based	20 (88)
NNRTI-based	3 (13)

[1]Two VL>50cp/mL [53cp/ml and 98 cp/ml]

Table S3: Study participant characteristics included in Figure 2

	Distribution of of integrated HIV DNA in CD4+ T cell subsets
	ART-suppressed
	n=14
Age, Years, median [IQR]	2.1 [1.4-3.4]
Gender, Female, n (%)	6 (43)
CD4 counts, cells/µl, median [IQR]	1627 [2000-2970]
CD8 counts, cells/µl, median [IQR]	2093 [1527-2583]
Ratio CD4/CD8, median [IQR]	1.16 [0.9-1.33]
VL <50 HIV RNA copies/ml, n (%)	13 (93)[1]
VL, Log10 (copies/ml of plasma)	
HIV transmission, n (%)	
In utero	5 (36)
Peripartum	2 (14)
Unknown	7 (50)
Prophylactic ARV history, n (%)	
None	2 (14)
AZT	5 (36)
AZT+3TC+NVP	7 (50)
Direct transition from prophylactic ARV to ART, n (%)	4 (29)
Duration prophylactic ARV, days, median [IQR]	28 [28-32]
Time between end of prophylactic ARV and ART initiation, days, median [IQR]	51 [1.0-58]
Time on ART, years, Median [IQR]	2.0 [1.3-3]
Age of ART initiation, weeks, median [IQR]	11.2 [5.2-12.6]
ART regimen, n (%)	
PI-based	13 (93)
NNRTI-based	1 (7)

[1]In one participant, VL was 98 HIV RNA copies/ml

Table S4: Study participant characteristics included in Figure S3

	Distribution of of integrated HIV DNA in CD4 T-cell subsets and inducible reservoir (HIV-Flow)<br>Viral failures<br>n=11
Age, Years, median [IQR]	1.92 [1.0-2.0]
Gender, Female, n (%)	3 (27)
CD4 counts, cells/µl, median [IQR]	1541 [1353-1774]
CD8 counts, cells/µl, median [IQR]	2414 [1565-2978]
Ratio CD4/CD8, median [IQR]	1.1 [0.5-1.2]
VL, Log_{10} (copies/ml of plasma)	4.83 [4.0-5.2]
HIV transmission, n (%)	
In utero	5 (45)
Peripartum	2 (18)
Unknown	4 (36)
Prophylactic ARV history, n (%)	
AZT	3 (27)
AZT+NVP	1 (9)
AZT+3TC+NVP	7 (64)
Direct transition from prophylactic ARV to ART, n (%)	7 (64)
Duration prophylactic ARV, days, median [IQR]	34 [30-40]
Time between end of prophylactic ARV and ART initiation, days, median [IQR]	1.0 [0.0-10]
Time on ART, years, Median [IQR]	1.9 [1.0-2.0]
Age of ART initiation, weeks, median [IQR]	5.4 [4.6-7.3]
ART regimen, n (%)	
PI-based	11 (100)
NNRTI-based	0 (0)

Table S5: Study participant characteristics included in Figure 3

	Evolution of the HIV persistence markers and distribution of integrated HIV DNA in CD4+ T-cell Subsets<br>n = 8
Gender, Female, n (%)	4 (50)
HIV transmission, n (%)	
In utero	3 (38)
Peripartum	2 (25)
Unknown	3 (38)
Prophylactic ARV history, n (%)	
None	0 (0)
AZT	3 (38)
AZT+3TC+NVP	5 (62)
Direct transition from prophylactic ARV to ART, n (%)	3 (38)
Duration prophylactic ARV, days, median [IQR]	29 [28-32]
Time between end of prophylactic ARV and ART initiation, days, median [IQR]	52 [0.8-58]
Age of ART initiation, weeks, median [IQR]	11.4 [4.3-13.1]
Age at T1, years median [IQR]	1.7 [1.2-2.5]
Age at T2, years median [IQR]	4.3 [3.1-5.0]
VL at V1 <50 HIV RNA copies/ml, n(%)	7 (88)[1]
VL at V2 <50 HIV RNA copies/ml, n(%)	8 (100)
CD4+ counts at T1, cells/µl, median [IQR]	2969 [2424-3193]
CD8+ counts at T1, cells/µl, median [IQR]	2098 [1614-2875]
Ratio CD4/CD8 at T1, median [IQR]	1.3 [1.1-1.7]
CD4+ counts at T2, cells/µl, median [IQR]	1911 [1665-2141]
CD8+ counts at T2, cells/µl, median [IQR]	1300 [1050-1858]
Ratio CD4/CD8 at T2, median [IQR]	1.2 [1.1-1.4]
Time on ART at T1, years, median [IQR]	1.6 [1.0-2.2]
Time on ART at T2, years, median [IQR]	4.2 [2.9-4.7]
ART regimen at T1, n (%)	
PI-based	8 (100)
NNRTI-based	0 (0)
ART regimen at T2, n (%)	
PI-based	5 (62)
NNRTI-based	3 (38)

[1]VL = 98 copies/ml;

Table S6: Study participant characteristics included in Figure 4

	**Longitudinal n = 49**
Gender, female, n (%)	23 (47)
HIV transmission, n (%)	
In utero	15 (31)
Peripartum	19 (39)
Unknown	15 (31)
Prophylactic ARV history, n (%)	
None	3 (6)
AZT	9 (18)
AZT+3TC+NVP	37 (76)
Direct transition from prophylactic ARV to ART	20 (41)
Duration prophylactic ARV, days, median [IQR]	33 [28-44]
Time between end of prophylactic ARV and ART initiation, days, median [IQR]	25 [0-61]
Age of ART initiation, months, median [IQR]	2.3 [1.2-3.4]
Age at first study visit, years median [IQR]	1 [0.2-1.3]
Age at last study visit, years median [IQR]	5 [2.8-7.4]
Continuous suppresion, <50 HIV RNA copies/ml, n(%)	50 (100)
Time on ART at first study visit, years, median [IQR]	0.9 [0-1.1]
Time on ART at last study visit, years, median [IQR]	4.9 [2.7-7.3]
Longitudinal follow up:	
>5 years on ART, n (%)	25 (50)
>6 years on ART, n (%)	18 (36)
>7 years on ART, n (%)	14 (28)
>8 years on ART, n (%)	10 (20)
>9 years on ART, n (%)	7 (14)
ART regimen, First Visit	
PI-based	46 (94)
NNRTI-based	3 (6)

Table S7: Study participant characteristics included in Figures 5 and 6

	0 year N=3	2 years N=6	3 years N=5	5-6 years N=5	>7 years N=7
Gender, Female, n (%)	2 (67)	2 (33)	2 (40)	2 (40)	3 (43)
HIV transmission, n (%)					
In utero	1 (33)	3 (19)	1 (20)	0 (0)	1 (14)
Peripartum	1 (33)	3 (19)	1 (20)	1 (20)	1 (14)
Unknown	1 (33)	10 (63)	3 (60)	4 (80)	5 (71)
Prophylactic ARV history, n (%)					
None	0 (0)	0 (0)	2 (40)	2 (40)	1 (14)
AZT	1 (33)	1 (17)	1 (20)	0 (0)	2 (29)
AZT+3TC+NVP	2 (67)	5 (83)	2 (40)	2 (40)	4 (57)
Unknown	0 (0)	0 (0)	0 (0)	1 (20)	0 (0)
Direct transition from prophylactic ARV to ART, n (%)	0 (0)	2 (33)	1 (20)	0 (0)	1 (14)
Duration prophylactic ARV, days, median [IQR]	42 [26-108]	42 [35-44]	33 [0-42]	21 [0-43]	42 [26-44]
Time between end of prophylactic ARV and ART initiation, days, median [IQR]	27 [16-43]	21 [5-49]	46 [16-67]	57 [41-81]	67 [37-83]
Age of ART initiation, weeks, median [IQR]	NA	9.1 [7-4-13.1]	9.6 [8.3-12.6]	12.6 [9.9-17.4]	11.9 [8.6-17.1]
Age, years median [IQR]	0.19 [0.16-0.37]	2.3 [2.3-2.4]	3.2 [3.1-3.4]	5.2 [5.2-5.6]	9.3 [8.0-9.7]
VL, log10, copies/ml, median [IQR]	6.3 [6.2-6.6]	-			
VL <50 HIV RNA copies/ml, n(%)	0 (0)	6 (100)	5 (100)	5 (100)	5 (100)
CD4+ counts, cells/µl, median [IQR]	NA	2173 [1789-3054]	2478 [2101-2626]	1183 [116-2003]	1160 [889-1390]
CD8+ counts, cells/µl, median [IQR]	NA	2005 [1240-3030]	2073 [1518-2664]	1547 [1430-1639]	998 [899-1297]
Ratio CD4/CD8, median [IQR]	NA	1.5 [1.0-1.6]	1.0 [0.9-1.2]	1.0 [0.8-1.3]	1.0 [0.9-1.1]
Time on ART, years, median [IQR]	-	2.1 [2.1-2.1]	3.0 [3.0-3.0]	5.0 [4.9-5.4]	9.1 [7.7-9.3]
ART regimen, n (%)					
PI-based	-	5 (83)	5 (100)	4 (80)	1 (14)
NNRTI-based	-	1 (17)	0 (0)	1 (20)	1 (14)
INSTI-based	-	-	-	-	5 (71)

Table S8: Antibodies used in this study

Target	Fluorochrome	Brand	Clone	Catalogue #
CD3	AF700	BD Biosciences	UCHT1	557943
CD4	APC	BD Biosciences	SK3	566915
CD4	BUV496	BD Biosciences	SK3	564651
CD8	PerCP-Cy5.5	BD biosciences	RPA-T8	560662
CD8	BUV395	BD Biosciences	RPA-T8	563795
CD14	V450	BD biosciences	MφP9	560349
CD69	PE-Cy7	BD biosciences	FN50	557745
CD4	FITC	BD biosciences	RPA-T4	561842
CD45RA	BV786	BD biosciences	HI100	563870
CD27	APC-eF780	Thermofisher	O323	47-0279
CD27	BV421	BD biosciences	L128	568226
CCR7	PE-CF594	BD Biosciences	150503	562381
CCR7	BB700	BD biosciences	3D12	566437
p24 KC57	PE	Beckman Coulter	KC57	6604667
p24 28B7	APC	MediMabs	28B7	MM-0289-APC
CD57	APC	BD Biosciences	NK-1	560845
CD95	PE-Cy7	BD Biosciences	DX2	561633
CD58	BV605	BD Biosciences	1C3	564362
HLA-DR	PerCP-Cy5.5	BD Biosciences	L243	339194
CD38	PE	BD Biosciences	HIT2	555460
PD-1	BUV 737	BD Biosciences	EH12.1	565299
CD31	BV421	BD Biosciences	WM59	564089

Figure S1

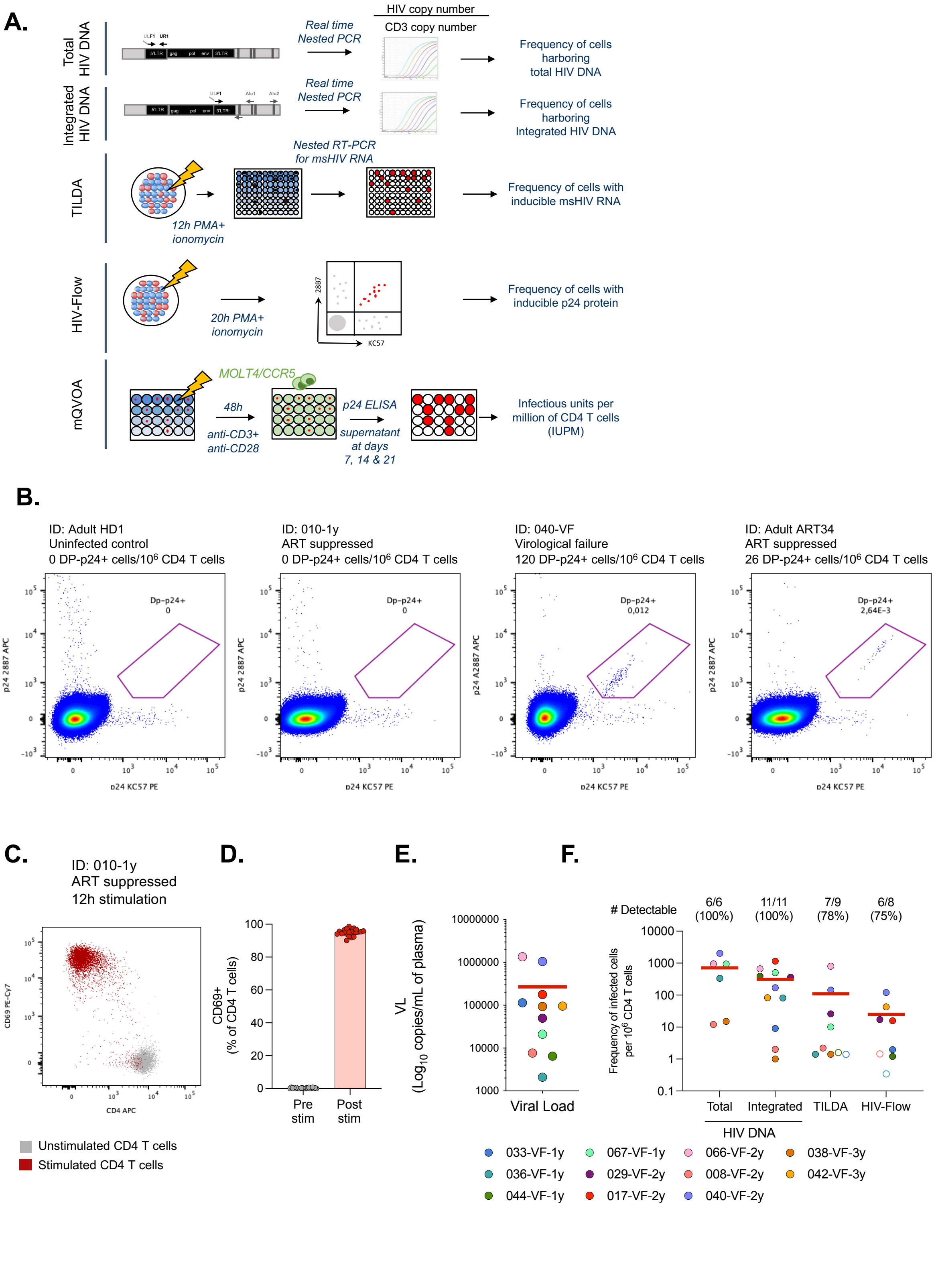

Figure S1: Experimental set up to measure markers of HIV persistence. A. Frequencies of infected cells were measured using five different assays. The frequencies of cells harboring total and integrated HIV DNA were quantified using a nested RT-qPCR *(50)*. The frequency of cells producing multiple spliced RNA (tat-rev) following 12 hours of PMA/ionomycin

stimulation were quantified by TILDA *(51)*. The frequency of cells expressing inducible p24 protein after 20h stimulation with PMA/ ionomycin was assessed by HIV-Flow *(39)*. Replication competent virus was evaluated using mQVOA*(52)*, which determines the infectious units per million of CD4+ T cells (IUPM). **B**. Representative dot plots showing the gating strategy of HIV-Flow used to analyze p24+ cells following 20 hours stimulation of CD4+ T cells with PMA/ionomycin. From left to right, the four plots display: 1) a control sample from an HIV-negative (uninfected) donor, 2) a sample from an ART-suppressed child, 3) a sample from a viremic child, and 4) a sample from an adult with HIV on ART. **C**. Representative dot plot showing the CD4+ T cells before (in grey) and after 20h stimulation with PMA/ionomycin (in red). **D**. CD69 expression in CD4+ T cells and after stimulation with PMA/ionomycin. There is a clear upregulation of CD69 marker and the downregulation of CD4 marker after stimulation in all samples. **E-F.** Viral load (**E**) and frequencies of infected cells (**F**) in samples from 11 early treated Thai children in virologic failure were estimated using up to four different assays: total HIV DNA (N=7), integrated HIV DNA (N=11), TILDA (N=9), HIV-Flow (N=8), depending on cell availability. The total number of detectable samples and corresponding frequencies are indicated. Red lines represent medians. Each sample is represented by a unique color-coded symbol and unique ID, followed by VF, which indicates virologic failure. Undetectable measurements are represented as open symbols, and the limits of detection are plotted.

Figure S2

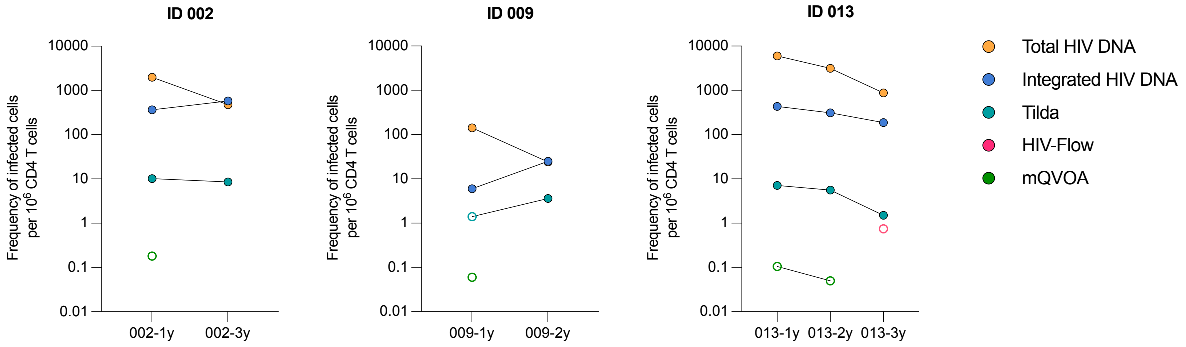

<u>Figure S2</u>: Evolution of HIV persistence markers in 3 participants followed longitudinally. Changes over time in the frequencies of infected cells in samples from 3 ART-suppressed children were assessed at two or three time points using four different assays: total HIV DNA, integrated HIV DNA, TILDA, HIV-Flow and/or mQVOA.

Figure S3

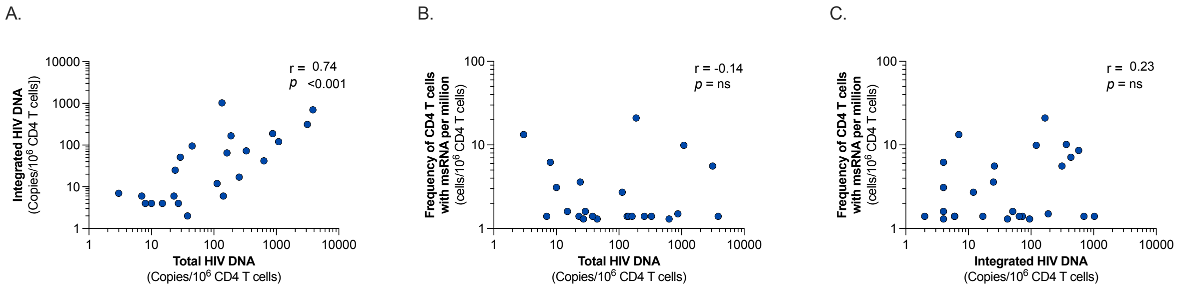

Figure S3: Correlation between markers of HIV persistence. Spearman correlation analyses were performed to compare the frequencies of infected cells measured using different techniques in 27 ART-suppressed children (same cohort as Figure 1). Panels show the correlations between: **(A)** total HIV DNA and integrated HIV DNA, **(B)** total HIV DNA and TILDA, and **(C)** integrated HIV DNA and TILDA. Each data point represents an individual, and correlation coefficients (Spearman's rho) and p-values are provided for each comparison.

Figure S4

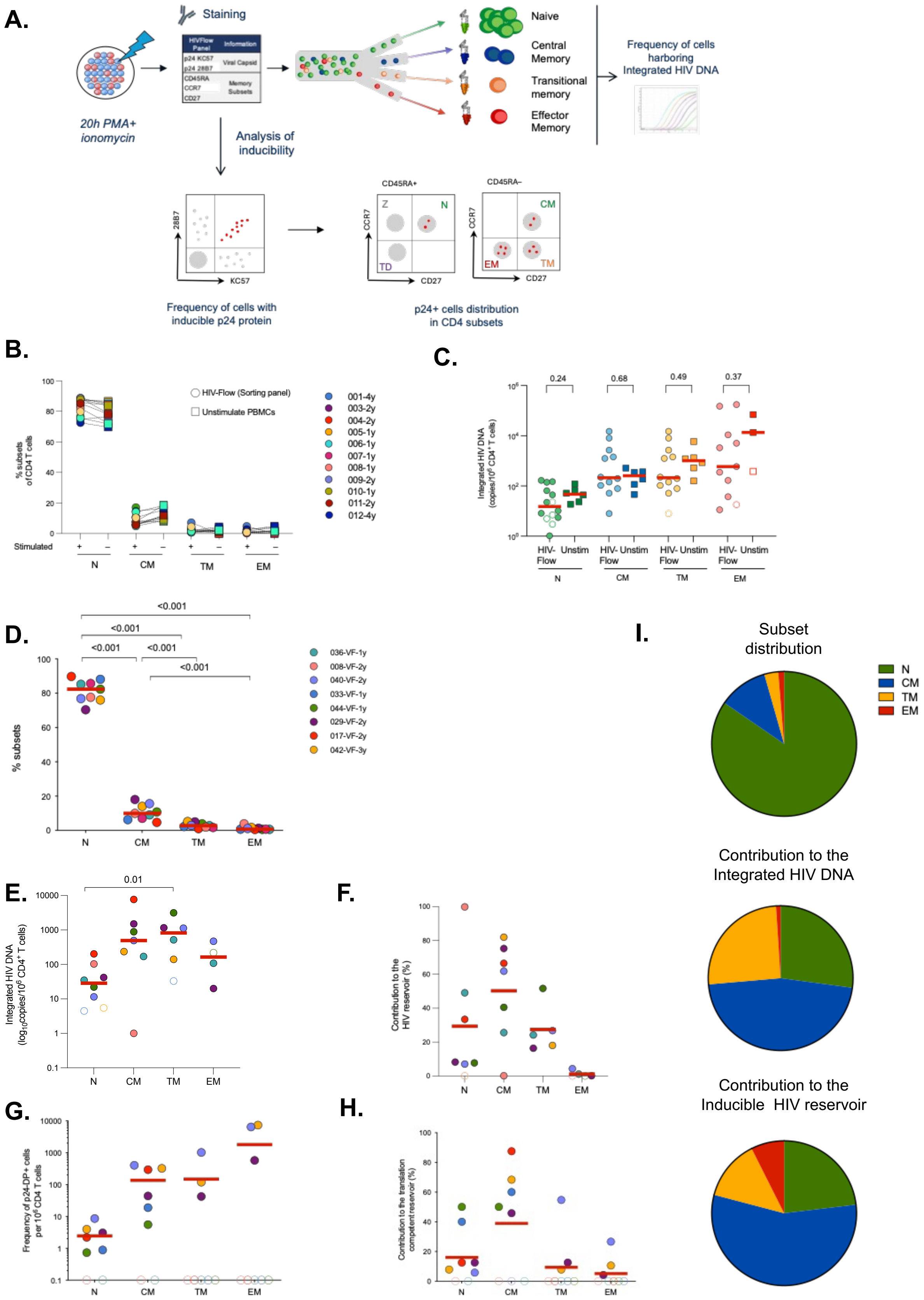

<u>Figure S4</u>: HIV-Flow Analysis and Characterization of CD4+ T cell Subsets in Early ART-Treated Thai Children with Virologic Failure A. HIV-Flow and cell-sorting protocol. Enriched CD4+ T cells were stimulated for 20h with PMA/Ionomycin in presence of brefeldin A. Then, a HIV-Flow staining was performed to determine CD4+ T-cell subset distribution as well as the number of cells expressing p24 protein. Stained cells were sorted to obtain naïve (CD45RA+ CCR7+ CD27+), central memory (CM, CD45RA- CCR7+CD27+), transitional memory (TM, CD45RA-CCR7-CD27+) and effector memory (EM, CD45RA-CCR7-CD27-). An additional minor population labeled "Z" (CD45RA+CCR7+CD27-) represents a small subset of naïve cells that lost CD27 expression following PMA/ionomycin stimulation. Integrated HIV DNA was quantified in each sorted subset using a nested RT-qPCR. **B**. Comparison of the frequency of CD4+ T cell subsets in paired samples (n=11) analyzed after PMA/ionomycin stimulation for HIV-Flow (Fig. 2) and in unstimulated PBMCs, showing that short-term stimulation does not substantially alter the distribution of naïve and memory CD4+ T cell subsets. **C**. Frequency of cells harboring integrated HIV DNA within CD4+ T cell subsets quantified either by HIV-Flow following PMA/ionomycin stimulation or by direct sorting of CD4+ T cell subsets, showing similar distributions across subsets with both approaches. **D**. Percentage of CD4+ T-cell subsets (Naïve, Central memory [CM], transitional memory [TM] and effector memory [EM]) among total CD4+ T-cells in 7 early treated Thai children in virologic failure. **E**. Frequency of cells harbouring integrated HIV DNA in sorted CD4+ T-cell subsets from the same individuals. Undetectable measurements are represented as open symbols. **F**. Contribution of each CD4+ T-cell subset to the pool of HIV-infected cells from each individual. **G**. Frequency of cells expressing p24 protein in each CD4+ T-cell subset. **H.** Contribution of each CD4+ T-cell subset to the pool of HIV-infected cells expressing p24 protein (inducible and translation-competent reservoir) from each individual. For panels B-F**,** red lines represent medians. Each sample is represented by a unique color-coded symbol and unique ID, followed by VF (virologic failure). **I**. Comparison of the contribution of the percentage of CD4+ T-cell subsets to the pool of circulating CD4+ T cells with the contribution of the each CD4+ T-cell subset to the pool of HIV-infected cells and to the inducible reservoir, summarized by pie charts displaying the median proportions across individuals.

Figure S5

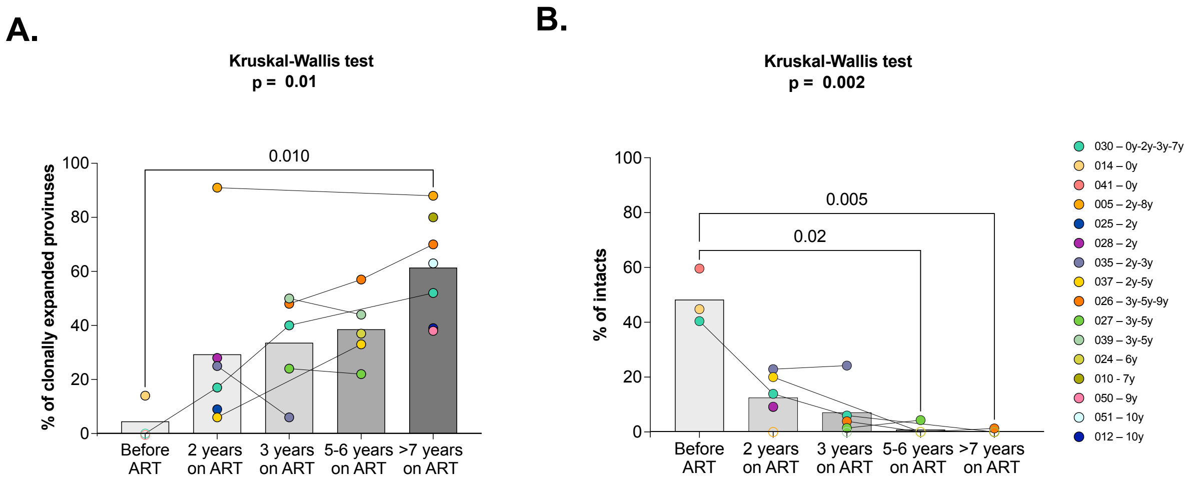

Supplementary Figure 5: Evolution of clonal expansion and intact proviruses over time in early ART-treated Thai children. A. Percentage of the HIV reservoir that is composed of clonally expanded proviruses for each time point. Each dot represents the percentage for each sample (see legend), and the bar graph corresponds to the mean value for the time point. P-value (Kruskal-Wallis test) indicates that the trend of an increasing proportion of clonal HIV sequences is significative (p=0.01). **B.** Percentage of the HIV reservoir that is composed of intact proviruses for each time point. Each dot represents the percentage for each sample (see legend), and the bar graph corresponds to the mean value for the time point. P-value (Kruskal-Wallis test) indicates that the trend of a decreasing proportion of intact HIV sequences is significative (p=0.002). Open-circle indicates that the value was undetectable.
